# Investigation of Potential cGMP-Specific PDE V and Aminopeptidase N Inhibitors of *Allium ampeloprasum* L. and Its Bioactive Components: Kinetic and Molecular Docking Studies

**DOI:** 10.3390/ijms241713319

**Published:** 2023-08-28

**Authors:** Jun-Hui Choi, Seung-Man Park, Seung Kim

**Affiliations:** 1 Department of Health Functional Food, Gwangju University, Gwangju 61743, Republic of Korea; sekai0572@naver.com; 2 Hyundai F&B Co., Ltd., Gwangju 61200, Republic of Korea

**Keywords:** *Allium ampeloprasum* L., aminopeptidase N, cGMP-specific PDE V, kinetics, molecular docking

## Abstract

The primary objectives of this study were to assess the inhibitory effects of *Allium ampeloprasum* L. extract (AAE) and its derived organosulfur and polyphenolic compounds on the enzymatic activities of cGMP-specific PDE V (PDE5) and aminopeptidase N (APN). Additionally, the study aimed to investigate their potential as inhibitors against these two target enzymes through kinetic analyses and molecular docking studies. The in vitro enzyme assays demonstrated that both AAE and its derived compounds significantly decreased the activity of PDE5 and APN. Further analyses involving kinetics and molecular docking provided insights into the specific inhibitor types of AAE and its derived compounds along with the proposed molecular docking models illustrating the interactions between the ligands (the compounds) and the enzymes (PDE5 and APN). In particular, AAE-derived polyphenolic compounds showed relatively stable binding affinity (−7.2 to −8.3 kcal/mol) on PDE5 and APN. Our findings proved the potential as an inhibitor against PDE5 and APN of AAE and AAE-derived organosulfur and polyphenolic compounds as well as a functional material for erectile dysfunction improvement.

## 1. Introduction

Male menopause is mainly caused by a decrease in testosterone and body aging, resulting from abnormal changes in the endocrine system [1,2]. Male menopausal symptoms include not only sexual function decline closely related to the decrease in testosterone but also decrease in sperm production, increase in insulin resistance, increase in visceral fat, decrease in physical function due to decrease in muscle mass and bone density, decrease in cognitive ability and spatial perception, fatigue, nervousness, and depression [3,4]. The male menopausal symptoms have a considerable negative impact on the health-related quality of life, affecting physical, mental, and social aspects in men. As the elderly male population experiencing menopausal symptoms increases, it is anticipated that there will be a rise in health expenditure and the emergence of various social issues [5,6]. A representative symptom of sexual dysfunction is erectile dysfunction (ED), and a commonly used treatment is using oral type 5 phosphodiesterase (PDE5) inhibitors such as sildenafil [7] and neprilysin (NEP) inhibitor [8]. The activities and functions of the heart and blood vessels are regulated by the interaction between cardiokines and cardiomyokines, which are specific cardiomyocytes-derived peptides [9,10]. Natriuretic peptides (NPs) play a crucial role in the cardiac endocrine system by utilizing guanosine 3′,5′-cyclic monophosphate (cGMP) as a second messenger and interacting with PDEs. Atrial myocytes are responsible for synthesizing atrial natriuretic factor (ANF), which is also known as atrial natriuretic peptide (ANP) [9,10]. Although referred to as brain natriuretic peptide (BNP), it is primarily located in atrial cardiomyocytes [9,10]. The family of natriuretic peptides includes a third peptide called C-type natriuretic peptide (CNP). In healthy individuals, CNP is found at relatively low levels in the central nervous system, chondrocytes (cartilage cells), and the cardiovascular system [9,10]. The clearance of cardiac peptides involves two primary mechanisms. The first mechanism is a cellular process where the ligand-natriuretic peptide receptor C (NPR-C) complex is internalized through the cell membrane. This internalization is followed by lysosomal hydrolysis of the natriuretic peptide and subsequent recycling of the NPR-C back to the cell surface. The second mechanism involves an enzymatic process, wherein a cleavage proteolytic enzyme called NEP is responsible for the degradation of the peptides [10]. The inhibition of metalloproteases such as NEP and aminopeptidase A (APA) or N (APN) (EC 3.4.11.2) increases levels of natriuretic peptides, bradykinin, and adrenomedullin, thus resulting in vasodilatation. Moreover, these natriuretic peptides act at the heart level by stimulating the synthesis of cyclic nucleotides such as cGMP. PDEs, responsible for their hydrolysis, govern physiological and hormonal responses under normal and pathological conditions by controlling the signalosome at the phosphorylation cascades and the expression of genes dependent on cyclic nucleotides [10]. It is therefore important not to neglect this important path of cGMP regulation mediated by the NPs. In particular, PDE5 (EC 3.1.4.17) is found in the heart, vascular smooth muscle, and endothelium. A particular PDE5 inhibitor, sildenafil, has been employed to treat erectile dysfunction [11]. Clinical studies in humans have revealed the advantageous impact of PDE5 inhibitors in heart failure management [12] and the treatment of pulmonary arterial hypertension [13]. Therefore, the inhibition of NPs or cGMP hydrolysis/degradation has been suggested as a strong way to improve cardiac and endovascular vasodilation. The interruption of enzymatic activities of APN and PDE5 directly help to improve cGMP regulation and vasodilatation in cardiomyocytes, cardiac fibroblasts, endothelial cells, smooth muscle cells, and related tissues [10].

Giant garlic (*Allium ampeloprasum* L.), called elephant garlic or great-headed garlic, is large enough to weigh up to 450 g [14]. Compared to garlic, it has a milder flavor and is sweeter, so it is used as a substitute for garlic in many countries, and it has characteristics closer to leek than garlic [15]. The components of *Allium ampeloprasum* L. vary depending on the place of production or cultivation conditions, but it is generally known to contain a lot of water, minerals such as Ca, P, and Fe, and various sulfur compounds [16]. It has been reported that the white bulb of elephant garlic contains a large amount of polyphenols such as kaempferol (KAE), quercetin (QUE), and sulfur compounds such as isoalliin (IA), methiin (MT), diallyl trisulfide (DATS), and diallyl disulfide (DADS) [17,18,19]. A potential of *Allium ampeloprasum* L. for improving various symptoms of menopause involving androgenic effects, increasing testosterone production, reducing obesity, blood pressure, and cholesterol, and enhancing antioxidant effects have been reported [4,14,20,21,22,23,24], and they are being used for commercialization in the food material for men’s health and the related functional food market worldwide [14,25,26], but related scientific evidence is still lacking. We analyzed the inhibitory effect of APN and PDE5 on the bio-active substances diallyl trisulfide, diallyl disulfide, kaempferol, quercetin, methiin, and isoalliin contained in elephant garlic, *Allium ampeloprasum* L., and performed a kinetic study using various kinetic parameter and molecular docking analysis by the binding energy scores and simulation software. We now report for the first time the APN and PDE5 inhibitor properties of *Allium ampeloprasum* L.-derived active ingredients.

## 2. Results

### 2.1. Effects of AAE, DATS, DADS, KAE, QUE, MT, and IA on PDE5 Activity

**Figure 1:**
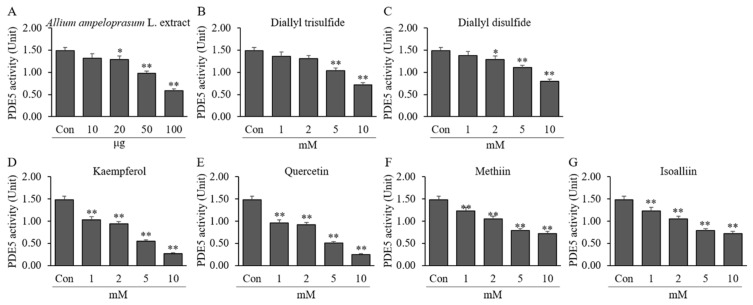
Effects of AAE and AAE-derived compounds on PDE5 activity. PDE5 was pretreated with AAE (0.01–0.1 mg) (**A**), DATS (**B**), DADS (**C**), KAE (**D**), QUE (**E**), MT (**F**), or IA (**G**) (1, 2, 5, and 10 mM) for 10 min. Following the addition of 10 mM p-NPPPh, the reaction mixture was incubated at 37 °C for 10 min. After the incubation period, the concentration of liberated 4-nitrophenol was determined by measuring its absorbance values at a wavelength of 405 nm using a microplate reader. Each value is the mean ± SD of triplicate measurements. * *p* < 0.05 and ** *p* < 0.01, compared to each control group (Con). Con, control group treated enzyme only.

The effect of AAE, DATS, DADS, KAE, QUE, MT, and IA on the enzymatic activity of PDE5 with p-NPPPh as a substrate is shown in Figure 1. The enzymatic activity of PDE5 was decreased upon treatment with 10, 20, 50, and 100 μg of AAE by 1.32 ± 0.09, 1.29 ± 0.08, 0.98 ± 0.05, 0.59 ± 0.04 U (10.97–60.63% reduction) compared to enzymatic activity without sample treatment (1.49 ± 0.07 U) (Figure 1A). As shown in Figure 1B–E, the enzymatic activity of PDE5 was decreased by treatment with 1, 2, 5, and 10 mM of DATS (8.34–51.75% reduction), DADS (7.40–46.57% reduction), KAE (30.89–82.10% reduction), QUE (35.40–83.18% reduction), MT (24.83–53.90% reduction), or IA (17.23–51.41% reduction). The IC_50_ values were found to be 80.53 μg (AAE), 9.49 mM (DATS), 10.75 mM (DADS), 3.94 mM (KAE), 3.49 mM (QUE), 7.56 mM (MT), and 8.42 mM (IA) for PDE5, respectively. These results from PDE5 assays indicate that AAE and AAE-derived active ingredients including organosulfur and polyphenolic compounds may attenuate the degradation of cGMP as secondary messengers by direct inhibition.

### 2.2. Effects of AAE, DATS, DADS, KAE, QUE, MT, and IA on APN Activity

**Figure 2:**
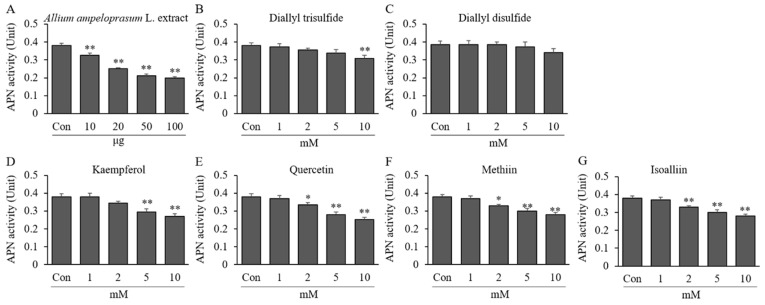
Effects of AAE and AAE-derived compounds on APN activity. The enzyme substrate, 40 μL of L-p-NA (2 mM), was added to 40 μL HEPES-buffer with or without AAE (0.01–0.1 mg) (**A**), DATS (**B**), DADS (**C**), KAE (**D**), QUE (**E**), MT (**F**), or IA (**G**) (1, 2, 5, and 10 mM). The reaction was started by adding 20 μL enzyme solution (1 mU) and incubated for 60 min at 37 °C. The absorbance of samples were measured at 405 nm to determine the formation of p-nitroaniline. Each value is the mean ± SD of triplicate measurements. * *p* < 0.05 and ** *p* < 0.01, compared to each control group (Con). Con, control group treated enzyme only.

The effect of AAE, DATS, DADS, KAE, QUE, MT, and IA on APN enzymatic activity with L-p-NA is shown in Figure 2. AAE at 10–100 μg reduced APN enzymatic activity by 0.33–0.20 U (14.51–47.57% reduction) compared to the activity without AAE treatment (0.38 ± 0.02 U). As shown in Figure 2B–E, the enzymatic activity of APN was decreased upon treatment with 10 mM of AAE, DATS, DADS, KAE, QUE, MT, and IA by 0.20 ± 0.01, 0.31 ± 0.02, 0.34 ± 0.02, 0.27 ± 0.01, 0.25 ± 0.01, 0.26 ± 0.02, and 0.28 ± 0.01 U (11.48–47.57% reduction) compared to the activity without sample treatment (0.38 U). The IC_50_ value was calculated to be 94.06 μg, 28.02 mM, 39.93 mM, 16.18 mM, 14.09 mM, 15.50 mM, and 19.29 mM for APN. This result indicates that the treatment of AAE and AAE-derived active ingredients including organosulfur and polyphenolic compounds directly inhibits the hydrolysis/degradation of natriuretic peptides such as ANF.

### 2.3. Effects of AAE, DATS, DADS, KAE, QUE, MT, and IA on Kinetic Parameters of Enzymes

**Figure 3:**
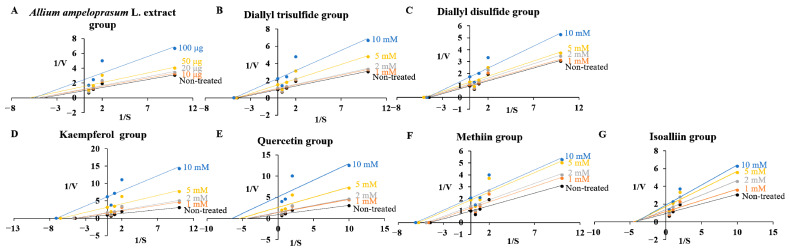
Kinetic and inhibitor studies with AAE (**A**), DATS (**B**), DADS (**C**), KAE (**D**), QUE (**E**), MT (**F**), and IA (**G**) for PDE5. Lineweaver–Burk plots were employed to analyze the inhibition of the target enzyme. These plots were represented as 1/velocity (1/V) versus 1/substrate concentration (1/[S]) in the presence or absence of each sample (non-treated (black circle), 10 μg or 1 mM (orange circle), 20 μg or 2 mM (silver circle), 50 μg or 5 mM (yellow circle), and 100 μg or 10 mM (blue circle)) as an inhibitor against PDE5.

**Figure 4:**
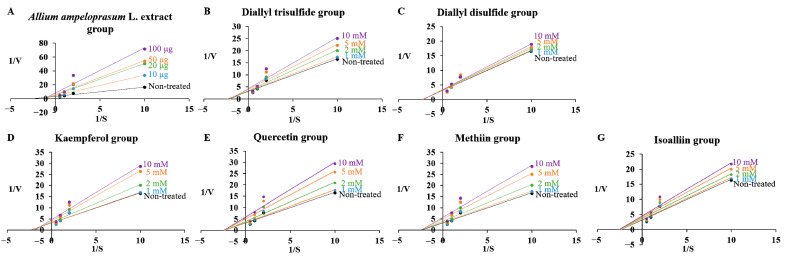
Kinetic and inhibitor studies with AAE (**A**), DATS (**B**), DADS (**C**), KAE (**D**), QUE (**E**), MT (**F**), and IA (**G**) for APN. Lineweaver–Burk plots were employed to analyze the inhibition of the target enzyme. These plots were represented as 1/velocity (1/V) versus 1/substrate concentration (1/(S)) in the presence or absence of each sample (non-treated (black circle), 10 μg or 1 mM (blue circle), 20 μg or 2 mM (green circle), 50 μg or 5 mM (orange circle), and 100 μg or 10 mM (purple circle)) as an inhibitor against APN.

The objective of this assay was to conduct further investigations on whether AAE and its active ingredients could act as inhibitors, displaying competitive, noncompetitive, uncompetitive, or mixed inhibition types against two target enzymes. The *K*_m_ values for each substrate were determined to be 0.223 ± 0.013 mM for PDE5 and 0.427 ± 0.017 mM for APN. The *K*_m_ value of PDE5 treated with the highest concentration of AEE and the compounds had significant differences by 0.63- (KAE), 0.69- (QUE), and 0.74-fold (MT) except for AAE, DATS, DADS, and IA in Table 1. The *K*_m_ value of APN treated with the highest concentration of samples had significant differences by 1.93- (AAE), 1.15- (DATS), and 1.24-fold (KAE), except for DADS, QUE, MT and IA in Table 2. The *V*_max_ values for PDE5 and APN were measured to be 0.316 ± 0.011 mU/min and 1.039 ± 0.052 mU/min, respectively. The *V*_max_ values of PDE5 at the highest concentration treatment of samples were decreased by 0.39- (AAE), 0.43- (DATS), 0.56- (DADS), 0.70- (KAE), 0.19- (QUE), 0.47- (MT), and 0.51-fold (IA) compared with the non-treated group (1.039 mU/min, 1.0-fold) in Table 1. The *V*_max_ values of APN at the highest concentration were decreased by 0.40- (AAE), 0.73- (DATS), 0.93- (DADS), 0.68- (KAE), 0.54- (QUE), 0.56- (MT), and 0.70-fold (IA) compared with the non-treated group (0.316 mU/min, 1.0-fold) in Table 2. The kinetic findings revealed that for PDE5, the reciprocal of *V*_max_ (1/*V*_max_) increased as the reciprocal of *K*_m_ (1/*K*_m_) increased in response to treatment with KAE, QUE, and MT, whereas for AEE, DATS, DADS, and IA, only 1/*V*_max_ increased without any changes in *K*_m_ (Figure 3). Additionally, the results indicated that on PDE5, treatment with AAE, DATS, and KAE led to an increase in both *K*_m_ and 1/*V*_max_ values, while for DADS, QUE, MT, and IA, only 1/*V*_max_ increased without any alterations in *K*_m_ (Figure 4) when considering APN as the target enzyme. These findings indicate that the enzymatic activity of PDE5 was inhibited by uncompetitive-type (KAE, QUE, and MT), and noncompetitive-type property (AEE, DATS, DADS, and IA) for PDE5, while the activity of APN was inhibited by mixed-type (AAE, DATS, and KAE), and noncompetitive-type property (DADS, QUE, MT, and IA) for PDE5 according to the putative decision system [27].

The catalytic rate constants of two target enzymes, *K*_cat_, were reduced through sample treatment, and *K*_i_ values of PDE5 and APN were calculated and then listed in Table 1 and Table 2. The enzyme inhibitory properties of AAE and AAE-derived active ingredients including organosulfur and polyphenolic compounds were analyzed using a Lineweaver–Burk plot (Figure 3 and Figure 4) and relative equations to estimate *K*_ik_ and *K*_iv_. The *K*_ik_, *K*_iv_, and *K*_ik_/*K*_iv_ ratio values of samples from PDE5 were determined to be 0.437, 0.065, and 6.8 (AAE), 133.4, 7.51, and 17.8 (DATS), 201.9, 12.61, and 16.0 (DADS), 16.92, 1.85, and 9.1 (KAE), 21.80, 2.32, and 9.4 (QUE), 28.02, 8.78, and 3.2 (MT), and 136.3, 10.46, and 13.0 (IA), respectively (Table 1). The values of three parameters from APN were determined to be 0.578 ± 0.352 mM, 0.052 ± 0.004 mM, 11.14 (AAE), 0.112 ± 0.008 μM, 0.052 ± 0.002 μM, 2.16 (DATS), 0.578 ± 0.352 mM, 0.052 ± 0.004 mM, 11.14 (DADS), 0.578 ± 0.352 mM, 0.052 ± 0.004 mM, 11.14 (KAE), 0.578 ± 0.352 mM, 0.052 ± 0.004 mM, 11.14 (QUE), 0.578 ± 0.352 mM, 0.052 ± 0.004 mM, 11.14 (MT), and 0.578 ± 0.352 mM, 0.052 ± 0.004 mM, 11.14 (IA), respectively (Table 2).

The *K*_ik_/*K*_iv_ ratio values for PDE5 inhibition in the MT group and APN inhibition in the DATS and IA groups were found to be 3.2, 2.5, and 4.8, respectively. These results are indicative of mixed-type inhibition. The *K*_ik_/*K*_iv_ ratio value of PDE5 inhibition in the DATS, DADS, KAE, QUE, and IA groups was 9.1–17.8, indicating that these compounds have noncompetitive-type inhibition characteristics. The *K*_ik_/*K*_iv_ ratio value of APN inhibition in the DADS, and KAE groups was 1.1–1.9, indicating that these compounds have an uncompetitive-type inhibition property, whereas the values of APN in the QUE and MT groups were observed to be 27.7 and 27.1, indicating that QUE and MT have a noncompetitive-type property according to the new decision system [27].

### 2.4. In Silico Molecular Docking Analysis

Molecular docking models of ligands and enzymes are presented in two different representations from AutoDockTools 1.5.6 (Figures S1A–S12A) and Discovery studio visualizer software (Figures S1C–S12C). The levels of hydrophobicity and the hydrogen bond in the binding pockets of all ligands are observed in Figure S1D,E–S12D,E. Among the AAE-derived active ingredients including organosulfur (DATS, DADS, MT, and IA) and polyphenolic (KAE and QUE) compounds on the PDE5 enzyme in Table 3 and Figures S1–S6, DATS and DADS interact with two amino acids of GLU536 and GLU539 located in the regulatory (R) domain of PDE5 in Figures S1A–E and S2A–E, resulting in an attractive charge of electrostatic interaction (GLU536, GLU539 for DATS or GLU539 for DADS) (Figure S1B) and van der Waals bond (GLU536 for DADS) and interacted with LEU540, ARG597, LEU600, and LYS604 to form van der Waals interactions (Figure S2B). KAE interacted with eight amino acids (GLU536, THR537, GLU539, LEU540, ARG597, LEU600, LYS604, and PRO696) of PDE5 in Figure S3A–E, and among them, GLU536 and THR537 in the R domain form a van der Waals bond, and GLU539 forms a conventional hydrogen bond. In addition, van der Waals bonds with LEU540 and PRO696, a conventional hydrogen bond with GLU597, and Pi-Alkyl of hydrophobic interaction bonds with ARG597, LEU600 and LYS604 were observed (Figure S3B). QUE interacted with seven amino acids (GLU539, ASP568, LYS603, LYS604, ASN605, TYR606, and LYS608) of PDE5 in Figure S4A–E, among which ASN605 forms a van der Waals, and LYS603, LYS604, TYR606, and LYS608 form conventional hydrogen bonds. The amino acids of GLU539, ASP568, and LYS608 were bound by electrostatic interactions (Pi-Anion and Pi-Cation), and LYS604 was bound by Pi-Alkyl of hydrophobic interaction (Figure S4B). MT and IA interacted with GLU539 of the R domain to form a conventional hydrogen bond and an attractive charge of electrostatic interaction (Figures S5A–E and S6A–E). MT interacted with GLU536, ARG597, LEU600, SER601, LYS604, ASN698 by van der Waals (Figure S5B), whereas IA interacted with GLU536, LEU540, ARG597, LEU600, SER601, LYS604, and ASN698 by forming van der Waals (Figure S6B). These docking results indicate that AAE-derived organosulfur and polyphenolic compounds bind to the R domain in PDE5, and it binds to several amino acids close to TYR612 in the core pocket (Q pocket) and HIS685 in a metal-binding site (M site).

Moreover, in the AAE-derived organosulfur (DATS, DADS, MT, and IA) and polyphenolic (KAE and QUE) compounds on the APN enzyme in Table 4 and Figures S7–S12, DATS interacted with nine amino acids (MET260, GLY261, ASN272, LYS274, TYR275, ILE751, ASN780, ARG783, and SER784) located in domains V–VII of the catalytic site of APN in Figure S7A–E; MET260, GLY261, ASN272, TYR275, ILE751, ASN780, and SER784 were bound by van der Waals, and LYS274 and ARG783 form an unfavorable positive-positive interaction (Figure S7B). DADS interacted with six amino acids (ARG349, PHE353, LYS541, GLU543, GLU627, and LEU628) located at the catalytic site of APN in Figure S8A–E; ARG349, LYS541, and LEU628 form van der Waals interactions, GLU627 forms a conventional hydrogen bond, GLU543 forms an attractive charge of electrostatic interaction, and PHE353 forms a Pi-sulfur bond (Figure S8B). KAE interacted with six amino acids (LYS286, LEU289, ASP290, ARG293, ASN343, and ASN344) in the catalytic site of APN in Figure S9A–E; amino acids of LEU289, ASN343, and ASN344 were bound by van der Waals, LYS286 and ARG293 form conventional hydrogen bonds, LYS286, ARG293, and ASN343 were bound by carbon hydrogen bonds, ASP290 forms a Pi-Anion of electrostatic interaction, and LYS286 forms a Pi-Alkyl of hydrophobic interaction (Figure S9B). QUE interacted with eight amino acids (LYS286, LEU289, ASP290, ARG293, ASN343, ASN344, ARG346, and GLU382) located in the catalytic site of APN in Figure S10A–E, among which the amino acids of LEU289, ARG293, ASN344, ARG346, and GLU382 form van der Waals interaction, conventional hydrogen bonds with LYS286 and ASN343, carbon hydrogen bonds with LYS286, ARG293, and ASN343, a Pi-Anion of electrostatic interaction with ASP290, a Pi-Cation of electrostatic interaction with LYS286, and a Pi-Sigma of hydrophobic interaction with LYS286 were observed (Figure S10B). MT interacted with three amino acids located at the catalytic site of APN in Figure S11A–E, among which VAL622 and THR755 form van der Waals interactions, and ARG641 forms an unfavorable positive–positive bond (Figure S11B). IA interacted with three amino acids located at the catalytic site of APN to form van der Waals bonds with ARG641, LEU752, and THR755 amino acids in Figure S12A–E. These docking results indicate that AAE-derived compounds bind to domain V–VII of the catalytic site of APN by interaction with MET260, GLY261, and ARG293, which partially coincide with the binding sites of bestatin and domain V–VII-specific residues.

In the binding modes (n = 9 or 10) of AAE-derived organosulfur and polyphenolic compounds for PDE5 analyzed by Autodock vina, the maximum binding affinities were −5.6 (DATS), −5.3 (DADS), −7.2 (KAE), −7.7 (QUE), −5.2 (MT), −7.1 (IA), and −9.4 kcal/mol (SILD), and the average binding affinity was −5.06, −4.74, −6.6, −6.0, −4.75, −5.16, and −8.54 kcal/mol in the above order (Table 5 and Figures S15–S20). In addition, the maximum binding affinities of APN binding modes (n = 9 or 10) were −5.4 (DATS), −5.0 (DADS), −8.1 (KAE), −8.3 (QUE), −5.9 (MT), −6.6 (IA), and −8.3 kcal/mol (BEST), and the average binding affinity was −4.59, −4.50, −7.59, −7.71, −5.05, −5.22, and −7.99 kcal/mol (Table 6 and Figures S21–S26). 

The results of Autodock vina show that all ligands showed relatively unstable binding affinities compared with the PDE5 inhibitor sildenafil on PDE5, whereas all ligands showed strong binding affinities of less than −5.0 on PDE5 and APN, and polyphenolics bind more stably to PDE5 and APN than organosulfur compounds.

## 3. Discussion

This study represents the first investigation to assess the inhibitory effects of AAE and AAE-derived compounds on PDE5 and APN as well as their molecular docking interactions and to characterize the inhibitor properties of these compounds against PDE5 and APN enzymes. As a result of comparing the results of in vitro enzyme assays, kinetic assays, and vina analysis of in silico molecular docking, the inhibition rate of PDE5 at the highest concentration in in vitro enzyme assays was confirmed: QUE (83%, IC50 = 3.49 mM) > KAE (82%, IC50 = 3.94 mM) > AAE (61%, IC50 = 80.53 μg) > MT (82%, IC50 = 3.94 mM) > DATS (52%, IC50 = 9.49 mM) > IA (51%, IC50 = 8.42 mM) > DADS (47%) in the order, and in kinetic assays, the *V*_max_ inhibition of PDE5 was listed: 0.19- (QUE) > 0.39- (AAE) > 0.43- (DATS) > 0.47- (MT) > 0.51- (IA) > 0.56- (DADS) > 0.70-fold (KAE). In the vina analysis, the maximum binding affinity of PDE5 was confirmed in the order of QUE (−7.7) > KAE (−7.2) > IA (−7.1) > DATS (−5.6) > DADS (−5.3) > MT (−5.2 kcal/mol). It shows that the inhibitory efficacy of polyphenolics as a PDE5 inhibitor is higher than that of sulfur-containing substances. The catalytic domain of the PDE5 molecule was divided into three subdomains: an N-terminal cyclin-fold domain (residues 537–678), a linker helical domain (residues 679–725) and C-terminal helical bundle domain (residues 726–860) [28]. The enzyme consists of four distinct subsites, namely, the M site, Q pocket, hydrophobic pocket (H pocket), and lid region (L region). Sildenafil as a PDE5 inhibitor has −9.4 kcal/mol of the maximum binding affinity and interacted with 11 amino acids (TYR612, LEU725, LEU765, ALA779, VAL782, ALA783, PHE786, LEU804, MET816, GLN817, and PHE820) located in the catalytic site of PDE5 by forming van der Waals, hydrogen bonds, unfavorable acceptor–acceptor, Pi-Sigma, Pi-Sulfur, Pi-Pi Stacked, Pi-Pi T-shaped, Alkyl, and Pi-Alkyl interactions shown in Table 3 and Figure S13. The Q pocket of the enzyme accommodates the pyrazolopyrimidinone group of sildenafil, which binds to the chemically similar guanidine group of cGMP in this specific region [28]. The pocket is formed by the residues TYR612, VAL782, GLN817, and PHE820, which are conserved to a high degree in PDE5. Additionally, the side chain of the pocket is stabilized through a hydrogen bond relay involving GLN817 to GLN775, GLN775 to ALA767, and GLN775 to TRP853. This arrangement facilitates the effective binding of sildenafil to the pocket. The relay mechanism plays a crucial role in determining the substrate specificity for cGMP in the pocket. It stabilizes the GLN817 side chain in an advantageous conformation, facilitating the formation of hydrogen bonds with the 6-CO and 1-NH groups on the purine base of cGMP [29]. Although AAE-derived compounds did not interact with the previously reported major catalytic site in PDE5 and thus did not form a critical bond that could affect activity, it was confirmed that the interaction of electrostatic, van der Waals, and hydrogen bonds with GLU539 in the R domain, which involve phosphorylation and cGMP binding ability [30], and ASN605 related to the Pγ C-terminus, and the catalytic pocket in complex with 3-isobutyl-1-methylxanthine (IBMX) [31] may affect the enzyme activity in all compounds. Comparing the results of in vitro enzyme assays, kinetic assays, and vina analysis of in silico molecular docking for APN, the results at the highest concentration were confirmed: AAE (48%) > QUE (33%) > MT (32%) > KAE (29%) > IA (26%) > DATS (19%) > DADS (11%) of in vitro enzyme assay; AAE (0.40-) > QUE (0.54-) > MT (0.56-) > KAE (0.68-) > IA (0.70-) > DATS (0.73-) > DADS (0.93-fold) of *V*_max_ inhibition in in vitro kinetic assay; −8.3 (BEST) > −8.3 (QUE) > −8.1 (KAE) > −6.6 (IA) > −5.9 (MT) > −5.4 (DATS) > −5.0 kcal/mol (DADS) of the maximum binding affinity in the vina analysis. The inhibitory efficacy of AAE was confirmed to be higher than that of other substances, and among substances, QUE showed the highest overall efficacy. In the APN structure, Domains V and VI encompass amino acids 253–580 and constitute the catalytic site responsible for the enzyme’s activity. These domains are vital as they serve as ligands for the zinc ion, which plays a crucial role in the enzymatic function. On the other hand, domain VII comprises amino acids 581 to 967 and represents the C-terminal of APN. It is the final domain containing a significant amount of alpha-helix content and facilitates dimerization through noncovalent bonding [32]. Bestatin as an APN inhibitor has −8.3 kcal/mol of the maximum binding affinity and interacted with 15 amino acids (GLN119, GLU121, MET260, GLY261, ALA262, MET263, GLU264, ARG293, VAL294, HIS297, GLU298, LYS319, GLU320, TYR376, TYR381) located in the catalytic site of APN by forming van der Waals, hydrogen bonds, unfavorable acceptor–acceptor, Pi-Sigma, Pi-Sulfur, Pi-Pi Stacked, Pi-Alkyl, and covalent bond interactions shown in Table 4 and Figure S14. DATS interacted with previously reported APN catalytic sites, MET260 and GLY261 in domains V and VI, and affected enzymatic activity, and it was revealed that KAE and QUE may affect the enzyme activity through strong affinity forming a hydrogen bond and van der Waals bond with ARG293 amino acid, which is the binding site of bestatin and actinonin [33]. Although the binding positions of MT and IA to APN did not coincide with those of bestatin, the two substances commonly interacted with residues ARG641 and THR755 to form hydrogen bonds and van der Waals bonds, indicating that MT and IA may affect enzyme activity by targeting domain VII in APN.

The PDE5 and APN inhibitory effects of elephant garlic-derived active ingredients have been reported in previous studies. In particular, organosulfur compounds as sulfur-containing substances including hydrogen sulfide, DATS, and DADS are known to act as H_2_S or NO donors [34,35]. In addition, it has been reported that hydrogen sulfide has sulfide post-translational modifications of eNOS and cGMP-dependent protein kinase 1α (PKG1α), and it forms 8-SH-cGMP from 8-nitro-cGMP, the soluble guanylate cyclase-β1 subunit (sGCβ1) persulfide (sGC-SSH) from sGCβ1, and PDE5 persulfide (PDE5-SSH) from PDE5 by electrophilic sulfhydration, eventually increasing cGMP levels and subsequent protein kinase G (PKG) activity [35]. However, studies on the effects of elephant garlic-derived organosulfur compounds DATS, DADS, MT, and IA on PDE5 and APN activities were not conducted, and no results were reported. An inhibitory effect against PDE5 has been reported in many polyphenolics and flavonoids, such as luteolin, kaempferol, quercetin, naringenin, taxifolin, aromadendrin, pelargonidin, and rutin, and this inhibitory action is known to be due to the unique structure of a C-2, C-3 double bond and the C-3 hydroxyl group of polyphenolics and flanovoids [36,37,38]. Previous studies have demonstrated that the inhibition of APN by flavonoids such as luteolin, quercetin, and 3′,4′-dihydroxyflavone does not necessitate hydroxylation at positions 5 and 7. However, it is essential for these flavonoids to undergo dihydroxylation at C-3′ and C-4′ and possess a double bond at positions C-2 and C-3 for exhibiting inhibitory activity [39]. The structural properties of flavonoids including 3,4-dihydroxyphenylacetic acid and 4-methylcatechol might be related to the inhibition of APN responsible for the splitting of regulatory neuropeptides [40]. Some docking analysis using quercetin with APN [41] showed that quercetin interacts with an energy of −6.9 kcal/mol with ALA474, ASP473, and SER476, suggesting the possible interaction and inhibition of APN by quercetin.

This study has certain limitations. Firstly, the experimental design in this research is confined to the in vitro level. Consequently, while the obtained results from in vitro enzyme assays, in vitro kinetics, and in silico molecular docking analysis can serve as a primary screening for inhibitory models against enzymatic activity, it is crucial to consider various factors at the in vivo biological level. For practical application, further optimization studies are necessary to develop a model that can be effective [42]. Secondly, it is essential to take into account the substrate concentration for enzymatic activity as well as the presence of co-factors and potential allosteric factors that may influence the activity of both inhibitors and enzymes [38]. Thirdly, follow-up studies focusing on the pharmaceutical or pharmacophore action, as well as the production process, are necessary to advance the development of inhibitors. Fourthly, in kinetic analysis, various components included in AEE can affect various factors including enzymatic activity and substrate, and the interfering effects of these various components do not support the validity of measurement and verification of kinetic parameters. Therefore, the enzymatic inhibitory effect of AEE using kinetic analysis should be utilized after separating and purifying specific components and substances present in AEE.

## 4. Materials and Methods

### 4.1. Materials

Trizma base, trizma HCl, aminopeptidase N (APN), L-leupine-p-nitroanilide (L-p-NA), cAMP-specific PDE V (PDE5), diallyl trisulfide (DATS), diallyl disulfide (DADS), kaempferol (KAE), quercetin (QUE), and p-nitrophenyl phenyl phosphonate (p-NPPPh) were purchased from Sigma-Aldrich (St. Louis, MO, USA). Methiin (MT) was purchased from Abcam (Cambridge, MA, USA). Isoalliin (IA) was purchased from DiagnoCine (Hackensack, NJ, USA). All other reagents used in this study were obtained commercially and were of high-quality grade.

### 4.2. Extraction of Allium ampeloprasum *L.*

Elephant garlic, *Allium ampeloprasum* L. grown in Uiseong-gun in Korea, was provided by Hyundai F&B Co., Ltd., Anseong-si, Republic of Korea, an agricultural corporation. *Allium ampeloprasum* L. was extracted by a previously described method [4]. The elephant garlic was peeled, washed with running water, and naturally dried. Then, 300 mL of 70% ethanol was added to 100 g of elephant garlic, and immersion extraction was performed at room temperature for 1 week. The extract was filtered with filter paper (Advantec, Tokyo, Japan) and concentrated using a rotary vacuum concentrator (Tokyo Rikakikai, Tokyo, Japan). The concentrated extract was prepared in a powder state using a freeze dryer (Ilshin Lab, Dongducheon, Korea), named as *Allium ampeloprasum* L. extract (AAE) stored at −20 °C, and used in the experiment. The yield of elephant garlic extract was calculated to be about 4.7% (4.7 g).

### 4.3. cGMP-Specific PDE V Assay

The specific substrate used for PDE5 was p-NPPPh, following a previously described method [43]. The enzyme solution was prepared by adding 1 U of PDE5 to a volume of 10 μL in 40 mM Tris-HCl buffer at pH 7.4. The substrate solution (10 mM) was prepared by dissolving the substrate in Tris-HCl buffer at pH 7.4. PDE5 was subjected to pretreatment with various substances, including AAE (0.01–0.1 mg), DATS, DADS, KAE, QUE, MT, or IA (1, 2, 5, and 10 mM), for a duration of 10 min. Following the pretreatment, the reaction was initiated and allowed to continue for an additional 10 min at 37 °C upon the addition of 10 mM p-NPPPh in a total reaction volume of 80 μL. Following the incubation period, the absorbance of the mixture was measured at 405 nm using a microplate reader. The enzyme activity was quantified in units, where one unit corresponds to the amount of enzyme needed to hydrolyze 1 micromol of p-NPPPh. The inhibitory activity of PDE5 (%) was calculated by determining the residual activity (U).

PDE5 inhibitory activity (%) = ([Residual unit _control without sample_ − Residual unit _control with sample_]/Residual unit _control without sample_) × 100

### 4.4. Aminopeptidase N Assay

The activity of APN was determined according to Melzig et al. [44]. Briefly, the enzyme substrate L-p-NA (2 mM) was dissolved in 50 mM N-(2-hydroxyethyl)piperazine-N’-2ethansulfonic acid (HEPES) buffer (pH 7.4) containing 154 mM NaCl. Aliquots of substrate (40 μL) were added to 40 μL HEPES-buffer with or without AAE (0.01–0.1 mg), DATS, DADS, KAE, QUE, MT, or IA (1, 2, 5, and 10 mM). The reaction was started by adding 20 μL enzyme solution (approximately 1 mU in HEPES-buffer) and incubated for 60 min at 37 °C. The samples were measured spectrophotometrically at 405 nm to determine the formation of p-nitroanilmne.

APN inhibitory activity (%) = ([Absorbance _control without sample_ − Absorbance _control with sample_]/Absorbance _control without sample_) × 100

### 4.5. Kinetic Assay

The kinetic constants were determined by constructing Lineweaver–Burk plots using the initial reaction rates obtained from various substrate concentrations (0.1, 0.5, 1, and 2 mM) for PDE5 as well as APN treated with or without substances such as AAE (0.01–0.1 mg), DATS, DADS, KAE, QUE, MT, or IA (1, 2, 5, and 10 mM). These reactions were conducted in a total volume of 80 μL. by a previously described method [45]. The Michaelis–Menten constant (*K*_m_) and maximal velocity (*V*_max_) were calculated using the x and y intercepts, respectively. Several catalytic rate constants (*K*_cat_) were determined by evaluating *V*_max_/(E_o_), where (E_o_) = 0.00013 μM (for PDE5) and 0.806 μM (for APN). The IC_50_ concentration, representing the concentration causing a 50% decrease in activity, was determined from activity (%) versus concentration plots for AAE, DATS, DADS, KAE, QUE, MT, or IA on the two target enzymes. Additionally, the *K*_ik_ and *K*_iv_ inhibition constants were calculated using previously described methods [27]. The inhibition type of AAE, DATS, DADS, KAE, QUE, MT, or IA as an inhibitor against the two target enzymes was determined based on previously described definitions for inhibition types [27].

### 4.6. Molecular Docking

Human phosphodiesterase 5A (PDB code: 1TBF), aminopeptidase N (PDB code: 2DQM), diallyl trisulfide (PubChem CID: 16315), diallyl disulfide (PubChem CID: 16590), kaempferol (PubChem CID: 5280863), quercetin (PubChem CID: 5280343), methiin (PubChem CID: 9578071), and isoalliin (PubChem CID: 5281112) were used for molecular docking analysis, and AutoDock4 [46] and UCSF Chimera [47] were used for analysis. The binding energy scores between the experimental ligands (AAE, DATS, DADS, KAE, QUE, MT, IA, sildenafil (SILD), and bestatin (BEST)) as an inhibitor and the proteins of PDE5 and APN were predicted using the AutoDock Vina software. AutoDock Vina is a molecular modeling and simulation software, and it is especially designed and effective for protein–ligand docking [48]. In the case of molecular binding analysis, grid maps including all residues were formed, and the number of evaluations was set to 2,500,000 and the number of runs was set to 10. Docking results were calculated using the Lamarckian Genetic Algorithm and visualized using AutoDockTools 1.5.6 and UCSF Chimera. AutoDockTools 1.5.6 and UCSF Chimera software (https://www.cgl.ucsf.edu/chimera/) were used to model the complex structure of the experimental ligands (AAE, DATS, DADS, KAE, QUE, MT, or IA) and the proteins of PDE5 and APN by the molecular docking within all amino acid residues located at each specific distance (Å). Images showing the binding pockets of enzymes and binding profiles with ligands were created using Discovery studio visualizer 2021 (v21.1.0.20298) software (Accelrys Software Inc., San Diego, CA, USA).

### 4.7. Statistical Analyses

Statistical analysis was carried out using SPSS 27 (27.0.0.0) software (SPSS Inc., Chicago, IL, USA). The data obtained and analyzed in this study were presented as mean ± standard deviation (SD). To determine statistical significance in multiple group comparisons, one-way analysis of variance (ANOVA) was employed, which was followed by a post hoc Tukey test. Statistical significance was defined as a *p*-value less than 0.05.

## 5. Conclusions

*Allium ampeloprasum* L. is a food material that is recently used for products for men’s health in Korea and worldwide, but there is a lack of research for scientific data and functional evaluation in the related functional field. Therefore, in the present study, PDE5 and APN inhibitory efficacy for ED improvement were evaluated at the in vitro and in silico levels using these AAE and AAE-derived compounds, and the molecular docking model of inhibitors targeting PDE5 and APN was suggested. AAE-derived organosulfur and polyphenolic compounds bind to the Glu539 of the R domain involved in phosphorylation and cGMP binding ability, and they bind to several amino acids close to TYR612 in the Q pocket and His685 in the M site of PDE5. Moreover, the AAE-derived compounds bind to domain V–VII of the catalytic site of APN by interaction with MET260, GLY261, and ARG293 and domain V–VII-specific residues. In particular, KAE and QUE in AAE-derived compounds showed relatively strong binding affinity and high enzyme inhibition activity on PDE5 and APN. Our findings proved the potential as an inhibitor against PDE5 and APN of AAE, and AAE-derived DATS, DADS, KAE, QUE, MT, and IA as well as a functional material for ED improvement.

## Figures and Tables

**Table 1:** Effect of AAE, DATS, DADS, KAE, QUE, MT, and IA on kinetic parameters of PDE5.

Compound		*K*_m_ (mM)	*V*_max_ (mU/min)	*K*_cat_ (k s^−1^)	*K*_i_	*K*_ik_	*K*_iv_	*K*_ik_/*K*_iv_
Non-treated		0.223 ± 0.013	1.039 ± 0.052	7858 ± 393	-	-	-	-
AAE (mg)	0.01	0.199 ± 0.011	0.899 ± 0.033	6795 ± 249	0.084 mg	0.437	0.065	6.8
	0.02	0.201 ± 0.015	0.849 ± 0.028	6415 ± 212				
	0.05	0.168 ± 0.011	0.646 ± 0.021	4887 ± 159				
	0.1	0.182 ± 0.012	0.408 ± 0.020	3085 ± 151				
DATS (mM)	1	0.211 ± 0.012	0.940 ± 0.026	7105 ± 197	7.53 mM	133.4	7.51	17.8
	2	0.206 ± 0.014	0.894 ± 0.032	6757 ± 242				
	5	0.221 ± 0.011	0.655 ± 0.023	4955 ± 174				
	10	0.208 ± 0.009	0.446 ± 0.019	3371 ± 144				
DADS (mM)	1	0.208 ± 0.010	0.959 ± 0.025	7253 ± 189	13.53 mM	201.9	12.61	16.0
	2	0.211 ± 0.013	0.876 ± 0.021	6625 ± 159				
	5	0.198 ± 0.013	0.781 ± 0.011	5905 ± 83				
	10	0.213 ± 0.012	0.580 ± 0.010	4383 ± 76				
KAE (mM)	1	0.215 ± 0.009	0.671 ± 0.013	5076 ± 98	3.61 mM	16.92	1.85	9.1
	2	0.214 ± 0.008	0.607 ± 0.010	4591 ± 76				
	5	0.153 ± 0.011	0.316 ± 0.016	2390 ± 121				
	10	0.140 ± 0.007	0.162 ± 0.009	1228 ± 69				
QUE (mM)	1	0.224 ± 0.016	0.724 ± 0.031	5473 ± 234	4.05 mM	21.80	2.32	9.4
	2	0.221 ± 0.011	0.686 ± 0.022	5187 ± 166				
	5	0.184 ± 0.015	0.383 ± 0.008	2893 ± 60				
	10	0.153 ± 0.009	0.195 ± 0.005	1478 ± 38				
MT (mM)	1	0.215 ± 0.010	0.828 ± 0.025	6261 ± 189	21.46 mM	28.02	8.78	3.2
	2	0.203 ± 0.014	0.738 ± 0.021	5580 ± 159				
	5	0.174 ± 0.014	0.530 ± 0.013	4007 ± 98				
	10	0.165 ± 0.011	0.486 ± 0.019	3673 ± 144				
IA (mM)	1	0.219 ± 0.012	0.871 ± 0.021	6587 ± 159	10.80 mM	136.3	10.46	13.0
	2	0.238 ± 0.015	0.726 ± 0.020	5489 ± 151				
	5	0.230 ± 0.013	0.580 ± 0.017	4387 ± 129				
	10	0.240 ± 0.011	0.531 ± 0.015	4018 ± 114				

*V*_max_ and *K*_m_ values were calculated according to Lineweaver–Burk from the data shown in Figure 3. The *K*_ik_/*K*_iv_ ratio was calculated according to Yang et al. [27]. Each value is the mean ± SD of triplicate measurements. DATS, diallyl trisulfide; DADS, diallyl disulfide; KAE, kaempferol; QUE, quercetin; MT, methiin; IA, isoalliin.

**Table 2:** Effect of AAE, DATS, DADS, KAE, QUE, MT, and IA on kinetic parameters of APN.

Compound		*K*_m_ (mM)	*V*_max_ (mU/min)	*K*_cat_ (s^−1^)	*K*_i_	*K*_ik_	*K*_iv_	*K*_ik_/*K*_iv_
Non-treated		0.427 ± 0.017	0.316 ± 0.011	392.0 ± 13.6	-	-	-	-
AAE (mg)	0.01	0.763 ± 0.026	0.254 ± 0.005	315.5 ± 6.2	0.057 mg	0.108	0.066	1.6
	0.02	0.884 ± 0.025	0.194 ± 0.006	240.3 ± 7.4				
	0.05	0.840 ± 0.021	0.171 ± 0.003	212.4 ± 3.7				
	0.1	0.822 ± 0.020	0.126 ± 0.003	156.3 ± 3.7				
DATS (mM)	1	0.422 ± 0.012	0.297 ± 0.006	368.9 ± 7.4	18.74 mM	0.685	0.269	2.5
	2	0.499 ± 0.014	0.294 ± 0.005	365.3 ± 6.2				
	5	0.466 ± 0.015	0.249 ± 0.004	309.2 ± 5.0				
	10	0.489 ± 0.011	0.230 ± 0.004	285.9 ± 5.0				
DADS (mM)	1	0.439 ± 0.013	0.318 ± 0.003	394.8 ± 3.7	65.71 mM	1.369	1.250	1.1
	2	0.437 ± 0.016	0.306 ± 0.006	379.5 ± 7.4				
	5	0.440 ± 0.012	0.297 ± 0.005	368.8 ± 6.2				
	10	0.461 ± 0.015	0.294 ± 0.004	364.9 ± 5.0				
KAE (mM)	1	0.441 ± 0.015	0.319 ± 0.006	396.3 ± 7.5	11.94 mM	0.421	0.217	1.9
	2	0.478 ± 0.011	0.284 ± 0.006	352.8 ± 7.4				
	5	0.563 ± 0.013	0.248 ± 0.004	308.0 ± 5.0				
	10	0.528 ± 0.014	0.216 ± 0.003	268.5 ± 3.7				
QUE (mM)	1	0.430 ± 0.011	0.297 ± 0.005	368.1 ± 6.2	13.19 mM	3.272	0.118	27.7
	2	0.442 ± 0.009	0.255 ± 0.006	316.6 ± 7.4				
	5	0.415 ± 0.010	0.197 ± 0.003	244.1 ± 3.7				
	10	0.414 ± 0.013	0.171 ± 0.002	212.3 ± 2.5				
MT (mM)	1	0.419 ± 0.012	0.296 ± 0.003	367.0 ± 3.7	13.43 mM	3.428	0.126	27.1
	2	0.411 ± 0.007	0.250 ± 0.005	310.6 ± 6.2				
	5	0.407 ± 0.009	0.199 ± 0.003	246.8 ± 3.7				
	10	0.415 ± 0.010	0.176 ± 0.002	218.9 ± 2.5				
IA (mM)	1	0.430 ± 0.011	0.307 ± 0.005	381.3 ± 6.2	33.08 mM	1.142	0.237	4.8
	2	0.395 ± 0.009	0.267 ± 0.006	331.6 ± 7.4				
	5	0.391 ± 0.013	0.241 ± 0.004	298.9 ± 4.9				
	10	0.392 ± 0.010	0.222 ± 0.003	275.8 ± 3.7				

*V*_max_ and *K*_m_ values were calculated according to Lineweaver–Burk from the data shown in Figure 4. The *K*_ik_/*K*_iv_ ratio was calculated according to Yang et al. [27]. Each value is the mean ± SD of triplicate measurements. DATS, diallyl trisulfide; DADS, diallyl disulfide; KAE, kaempferol; QUE, quercetin; MT, methiin; IA, isoalliin.

**Table 3:** Molecular interaction of PDE5 with AAE-derived compounds.

Ligand	Van der Waals	Hydrogen Bond	Electrostatic			Hydrophobic
DATS	LEU540, ARG597, LEU600, LYS604	-	Attractive charge: GLU536, GLU539	-	-	-
DADS	GLU536, LEU540, ARG597, LEU600, LYS604	-	Attractive charge: GLU539	-	-	-
KAE	GLU536, THR537, LEU540, PRO696	Conventional: GLU539, ARG597	-	-	-	Pi-Alkyl: ARG597, LEU600, LYS604
QUE	ASN605	Conventional: LYS603, LYS604, TYR606, LYS608	-	Pi-Anion: GLU539, ASP568	Pi-Cation: LYS608	Pi-Alkyl: LYS604
MT	GLU536, ARG597, LEU600, SER601, LYS604, ASN698	Conventional: GLU539	Attractive charge: GLU539	-	-	-
IA	GLU536, LEU540, ARG597, LEU600, SER601, LYS604, ASN698	-	Attractive charge: GLU539	-	-	-

(-) interaction, not detected. DATS, diallyl trisulfide; DADS, diallyl disulfide; KAE, kaempferol; QUE, quercetin; MT, methiin; IA, isoalliin.

**Table 4:** Molecular interaction of APN with AAE-derived compounds.

Ligand	Van der Waals	Hydrogen Bond	Unfavorable	Electrostatic			Hydrophobic		Miscellaneous
DATS	MET260 *, GLY261 *, ASN272, TYR275, ILE751, ASN780, SER784	-	Positive–positive: LYS274, ARG783	-	-	-	-	-	-
DADS	ARG349, LYS541, LEU628	Conventional: GLU627	-	Attractive charge: GLU543	-	-	-	-	Pi-Sulfur: PHE353
KAE	LEU289, ASN343, ASN344	Conventional: LYS286, ARG293 * Carbon hydrogen: LYS286, ARG293 *, ASN343	-	-	Pi-Anion: ASP290	-	Pi-Alkyl: LYS286	-	-
QUE	LEU289, ARG293 *, ASN344, ARG346, GLU382	Conventional: LYS286, ASN343 Carbon hydrogen: LYS286, ARG293 *, ASN343	-	-	Pi-Anion: ASP290	Pi-Cation: LYS286	-	Pi-Sigma: LYS286	-
MT	VAL622, THR755	-	Positive–positive: ARG641	-	-	-	-	-	-
IA	ARG641, LEU752, THR755	-	-	-	-	-	-	-	-

* Residues matching the binding site of bestatin. (-) interaction, not detected. DATS, diallyl trisulfide; DADS, diallyl disulfide; KAE, kaempferol; QUE, quercetin; MT, methiin; IA, isoalliin.

**Table 5:** Docking energy for the molecular binding of AAE-derived compounds on PDE5.

Ligand	Lowest Binding Energy (kcal/mol)	Average Docking Energy (kcal/mol)
DATS	−5.6	−5.06 (n = 10)
DADS	−5.3	−4.74 (n = 10)
KAE	−7.2	−6.6 (n = 9)
QUE	−7.7	−6.0 (n = 9)
MT	−5.2	−4.75 (n = 10)
IA	−7.1	−5.16 (n = 10)
SILD	−9.4	−8.54 (n = 9)

Docking energy was analyzed by Autodock Vina. n = numbers of binding modes. DATS, diallyl trisulfide; DADS, diallyl disulfide; KAE, kaempferol; QUE, quercetin; MT, methiin; IA, isoalliin; SILD, sildenafil.

**Table 6:** Docking energy for the molecular binding of AAE-derived compounds on APN.

Ligand	Lowest Binding Energy (kcal/mol)	Average Docking Energy (kcal/mol)
DATS	−5.4	−4.59 (n = 10)
DADS	−5.0	−4.50 (n = 10)
KAE	−8.1	−7.59 (n = 9)
QUE	−8.3	−7.71 (n = 9)
MT	−5.9	−5.05 (n = 10)
IA	−6.6	−5.22 (n = 10)
BEST	−8.3	−7.99 (n = 9)

Docking energy was analyzed by Autodock Vina. n = numbers of binding modes. DATS, diallyl trisulfide; DADS, diallyl disulfide; KAE, kaempferol; QUE, quercetin; MT, methiin; IA, isoalliin; BEST, bestatin.

## Data Availability

Not applicable.

## Supplementary Materials

The following supporting information can be downloaded at: https://www.mdpi.com/article/10.3390/ijms241713319/s1.

## References

[1] Decaroli M.C., Rochira V. (2017). Aging and sex hormones in males. Virulence.

[2] Dudek P., Kozakowski J., Zgliczyński W. (2017). Late-onset hypogonadism. Prz. Menopauzalny.

[3] Schubert M., Jockenhövel F. (2005). Late-onset hypogonadism in the aging male (LOH): Definition, diagnostic and clinical aspects. J. Endocrinol. Investig..

[4] Park S.E., Lee H.J., Jeong I.S., Kim S. (2022). Effects of elephant garlic (*Allium ampeloprasum*) extract on testosterone synthesis in TM3 Leydig cells. Korean J. Food Preserv..

[5] Wang C., Nieschlag E., Swerdloff R., Behre H.M., Hellstrom W.J., Gooren L.J., Kaufman J.M., Legros J.J., Lunenfeld B., Morales A. (2008). Investigation, treatment and monitoring of late-onset hypogonadism in males: ISA, ISSAM, EAU, EAA and ASA recommendations. Eur. J. Endocrinol..

[6] Singh P. (2013). Andropause: Current concepts. Indian J. Endocrinol. Metab..

[7] Sun T., Xu W., Tu B., Wang T., Liu J., Liu K., Luan Y. (2023). Engineered adipose-derived stem cells overexpressing RXFP1 via CRISPR activation ameliorate erectile dysfunction in diabetic rats. Antioxidants.

[8] Sert S., Karabay E., Gungor B., Yildirimturk O. (2023). Effect of angiotensin receptor-neprilysin inhibitor treatment on erectile dysfunction in heart failure with a reduced ejection fraction. Marmara Med. J..

[9] Srivastava H., Pozzoli M., Lau E. (2022). Defining the roles of cardiokines in human aging and age-associated diseases. Front. Aging.

[10] Lugnier C., Meyer A., Charloux A., Andrès E., Gény B., Talha S. (2019). The endocrine function of the heart: Physiology and involvements of natriuretic peptides and cyclic nucleotide phosphodiesterases in heart failure. J. Clin. Med..

[11] Boolell M., Allen M.J., Ballard S.A., Gepi-Attee S., Muirhead G.J., Naylor A.M., Osterloh I.H., Gingell C. (1996). Sildenafil: An orally active type 5 cyclic GMP-specific phosphodiesterase inhibitor for the treatment of penile erectile dysfunction. Int. J. Impot. Res..

[12] Guazzi M., Vicenzi M., Arena R., Guazzi M.D. (2011). PDE5 inhibition with sildenafil improves left ventricular diastolic function, cardiac geometry, and clinical status in patients with stable systolic heart failure: Results of a 1-year, prospective, randomized, placebo-controlled study. Circ. Heart Fail..

[13] Klinger J.R., Thaker S., Houtchens J., Preston I.R., Hill N.S., Farber H.W. (2006). Pulmonary hemodynamic responses to brain natriuretic peptide and sildenafil in patients with pulmonary arterial hypertension. Chest.

[14] Kim D., Kim K.H., Yook H.S. (2015). Analysis of active components of giant black garlic. J. Korean Soc. Food Sci. Nutr..

[15] Rattanachaikunsopon P., Phumkhachorn P. (2009). Antimicrobial activity of elephant garlic oil against *Vibrio cholerae* in vitro and in a food model. Biosci. Biotechnol. Biochem..

[16] Rabinowitch H.D., Brewster J.L. (1989). Onions and Allied Crops: Biochemistry Food Science Minor Crops.

[17] Satyal P., Craft J.D., Dosoky N.S., Setzer W.N. (2017). The chemical compositions of the volatile oils of garlic (*Allium sativum*) and wild garlic (*Allium vineale*). Foods.

[18] Shelke P.A., Rafiq S.M., Bhavesh C., Rafiq S.I., Swapnil P., Mushtaq R., Nayik G.A., Gull A. (2020). Leek (*Allium ampeloprasum* L.). Antioxidants in Vegetables and Nuts—Properties and Health Benefits.

[19] Polito F., Amato G., Caputo L., De Feo V., Fratianni F., Candido V., Nazzaro F. (2022). Chemical composition and agronomic traits of *Allium sativum* and *Allium ampeloprasum* leaves and bulbs and their action against *Listeria monocytogenes* and other food pathogens. Foods.

[20] Lee S.G., Hahn D.Y., Kim S.R., Lee W.Y., Nam J.O. (2020). Elephant garlic extracts inhibit adipogenesis in 3T3-L1 adipocytes. Microbiol. Biotechnol. Lett..

[21] Chae J., Lee E., Oh S.M., Ryu H.W., Kim S., Nam J.O. (2023). Aged black garlic (*Allium sativum* L.) and aged black elephant garlic (*Allium ampeloprasum* L.) alleviate obesity and attenuate obesity-induced muscle atrophy in diet-induced obese C57BL/6 mice. Biomed. Pharmacother..

[22] Dey P., Khaled K.L. (2013). An extensive review on *Allium ampeloprasum* a magical herb. Int. J. Sci. Res..

[23] Jalilian F., Chahardoli A., Sadrjavadi K., Fattahi A., Shokoohinia Y. (2020). Green synthesized silver nanoparticle from *Allium ampeloprasum* aqueous extract: Characterization, antioxidant activities, antibacterial and cytotoxicity effects. Adv. Powder Technol..

[24] Zhang F., Jia J., Yao X. (2023). *Allium ampeloprasum* leaf aqueous extract green-formulated Ag nanoparticles: Determination of anti-human lung cancer and antioxidant effects. J. Eng. Res..

[25] Shahrajabian M.H., Sun W., Cheng Q. (2021). A Review of Leek (*A. ampeloprasum* L.), an important vegetable and food ingredient with remarkable pharmaceutical activities. Pharmacogn. Commn..

[26] Cho Y.K., Ann S.W., Jang M.J., Oh T.S., Oh M.G., Park Y.J., Kim C.H. (2020). Analysis of biological activity by time of black garlic ripening in Seosan Yukjok garlic and elephant garlic. J. Environ. Sci. Int..

[27] Yang X., Du Z., Pu J., Zhao H., Chen H., Liu Y., Li Z., Cheng Z., Zhong H., Liao F. (2013). Classification of difference between inhibition constants of an inhibitor to facilitate identifying the inhibition type. J. Enzyme Inhib. Med. Chem..

[28] Sung B.J., Hwang K.Y., Jeon Y.H., Lee J.I., Heo Y.S., Kim J.H., Moon J., Yoon J.M., Hyun Y.L., Kim E. (2003). Structure of the catalytic domain of human phosphodiesterase 5 with bound drug molecules. Nature.

[29] Xu R.X., Hassell A.M., Vanderwall D., Lambert M.H., Holmes W.D., Luther M.A., Rocque W.J., Milburn M.V., Zhao Y., Ke H. (2000). Atomic structure of PDE4: Insights into phosphodiesterase mechanism and specificity. Science.

[30] Francis S.H., Bessay E.P., Kotera J., Grimes K.A., Liu L., Thompson W.J., Corbin J.D. (2002). Phosphorylation of isolated human phosphodiesterase-5 regulatory domain induces an apparent conformational change and increases cGMP binding affinity. J. Biol. Chem..

[31] Barren B., Gakhar L., Muradov H., Boyd K.K., Ramaswamy S., Artemyev N.O. (2009). Structural basis of phosphodiesterase 6 inhibition by the C-terminal region of the gamma-subunit. EMBO J..

[32] Luan Y., Xu W. (2007). The structure and main functions of aminopeptidase N. Curr. Med. Chem..

[33] Ganji R.J., Reddi R., Gumpena R., Marapaka A.K., Arya T., Sankoju P., Bhukya S., Addlagatta A. (2015). Structural basis for the inhibition of M1 family aminopeptidases by the natural product actinonin: Crystal structure in complex with E. coli aminopeptidase N. Protein Sci..

[34] Lei Y.P., Liu C.T., Sheen L.Y., Chen H.W., Lii C.K. (2010). Diallyl disulfide and diallyl trisulfide protect endothelial nitric oxide synthase against damage by oxidized low-density lipoprotein. Mol. Nutr. Food Res..

[35] Kolluru G.K., Shackelford R.E., Shen X., Dominic P., Kevil C.G. (2023). Sulfide regulation of cardiovascular function in health and disease. Nat. Rev. Cardiol..

[36] Sabphon C., Temkitthawon P., Ingkaninan K., Sawasdee P. (2015). Phosphodiesterase inhibitory activity of the flavonoids and xanthones from *Anaxagorea luzonensis*. Nat. Prod. Commun..

[37] Gaikwad D.T., Jadhav N.R. (2021). Discovery of potential inhibitors for phosphodiesterase 5A, sodium-potassium pump and beta-adrenergic receptor from *Terminalia arjuna*: In silico approach. J. Biomol. Struct. Dyn..

[38] Ganapathy A.A., Priya V.M.H., Kumaran A. (2021). Medicinal plants as a potential source of phosphodiesterase-5 inhibitors: A review. J. Ethnopharmacol..

[39] Parellada J., Guinea M. (1995). Flavonoid inhibitors of trypsin and leucine aminopeptidase: A proposed mathematical model for IC50 estimation. J. Nat. Prod..

[40] Bormann H., Melzig M.F. (2000). Inhibition of metallopeptidases by flavonoids and related compounds. Pharmazie.

[41] Manukyan A.E., Hovhannisyan A.A. (2021). Podophyllotoxin and quercetin in silico derivatives docking analysis with cyclooxygenase-2 and aminopeptidase-N. bioRxiv.

[42] Kim J.J., Moon D.G. (2008). Past, present and future of PDE5 inhibitor. Korean J. Androl..

[43] Ojo O.A., Ojo A.B., Oyinloye B.E., Ajiboye B.O., Anifowose O.O., Akawa A., Olaiya O.E., Olasehinde O.R., Kappo A.P. (2019). *Ocimum gratissimum* Linn. leaves reduce the key enzymes activities relevant to erectile dysfunction in isolated penile and testicular tissues of rats. BMC Complement. Altern. Med..

[44] Melzig M.F., Bormann H. (1998). Betulinic acid inhibits aminopeptidase N activity. Planta Med..

[45] Choi J.H., Kim S. (2022). In vitro antithrombotic, hematological toxicity, and inhibitor studies of protocatechuic, isovanillic, and p-hydroxybenzoic acids from *Maclura tricuspidata* (Carr.) Bur. Molecules.

[46] Morris G.M., Huey R., Lindstrom W., Sanner M.F., Belew R.K., Goodsell D.S., Olson A.J. (2009). AutoDock4 and AutoDockTools4: Automated docking with selective receptor flexibility. J. Comput. Chem..

[47] Pettersen E.F., Goddard T.D., Huang C.C., Couch G.S., Greenblatt D.M., Meng E.C., Ferrin T.E. (2004). UCSF Chimera—A visualization system for exploratory research and analysis. J. Comput. Chem..

[48] Sousa S.F., Fernandes P.A., Ramos M.J. (2006). Protein-ligand docking: Current status and future challenges. Proteins.

