# Supplementary material for: Investigation of Potential cGMP-Specific PDE V and Aminopeptidase N Inhibitors of Allium ampeloprasum L. and Its Bioactive Components: Kinetic and Molecular Docking Studies
Source: Int J Mol Sci. 2023 Aug 28;24(17):13319. doi: 10.3390/ijms241713319 (PMC10488055; doi:10.3390/ijms241713319)
Supplement: Supplementary file 1 [file ijms-24-13319-s001.zip › ijms-2589461-supplementary.pdf]

Supplementary Information for

Investigation of potential cGMP-specific PDE V and aminopeptidase N inhibitors of *Allium ampeloprasum* L. and its bioactive components: A kinetic and molecular docking studies

Jun-Hui Choi, Seung Kim*

Department of Health Functional Food, Gwangju University, Gwangju, 61743, Republic of Korea

Current file content:

• Figure S1. 3D molecular binding pattern (A) and 3D molecular interacting pattern (B,C) containing hydrogen bond pocket (D) and hydrophobic pocket (E) of DATS into domain R and the residues close to the catalytic site of PDE5.

• Figure S2. 3D molecular binding pattern (A) and 3D molecular interacting pattern (B,C) containing hydrogen bond pocket (D) and hydrophobic pocket (E) of DADS into domain R and the residues close to the catalytic site of PDE5.

• Figure S3. 3D molecular binding pattern (A) and 3D molecular interacting pattern (B,C) containing hydrogen bond pocket (D) and hydrophobic pocket (E) of KAE into domain R and the residues close to the catalytic site of PDE5.

• Figure S4. 3D molecular binding pattern (A) and 3D molecular interacting pattern (B,C) containing hydrogen bond pocket (D) and hydrophobic pocket (E) of QUE into domain R and the residues close to the catalytic site of PDE5.

• Figure S5. 3D molecular binding pattern (A) and 3D molecular interacting pattern (B,C) containing hydrogen bond pocket (D) and hydrophobic pocket (E) of MT into domain R and

the residues close to the catalytic site of PDE5.

• Figure S6. 3D molecular binding pattern (A) and 3D molecular interacting pattern (B,C) containing hydrogen bond pocket (D) and hydrophobic pocket (E) of IA into domain R and the residues close to the catalytic site of PDE5.

• Figure S7. 3D molecular binding pattern (A) and 3D molecular interacting pattern (B,C) containing hydrogen bond pocket (D) and hydrophobic pocket (E) of DATS into domain V-Vii of the catalytic site and domain V-Vii-specific residues of APN.

• Figure S8. 3D molecular binding pattern (A) and 3D molecular interacting pattern (B,C) containing hydrogen bond pocket (D) and hydrophobic pocket (E) of DADS into domain V-Vii of the catalytic site and domain V-Vii-specific residues of APN.

• Figure S9. 3D molecular binding pattern (A) and 3D molecular interacting pattern (B,C) containing hydrogen bond pocket (D) and hydrophobic pocket (E) of KAE into domain V-Vii of the catalytic site and domain V-Vii-specific residues of APN.

• Figure S10. 3D molecular binding pattern (A) and 3D molecular interacting pattern (B,C) containing hydrogen bond pocket (D) and hydrophobic pocket (E) of QUE into domain V-Vii of the catalytic site and domain V-Vii-specific residues of APN.

• Figure S11. 3D molecular binding pattern (A) and 3D molecular interacting pattern (B,C) containing hydrogen bond pocket (D) and hydrophobic pocket (E) of MT into domain V-Vii of the catalytic site and domain V-Vii-specific residues of APN.

• Figure S12. 3D molecular binding pattern (A) and 3D molecular interacting pattern (B,C) containing hydrogen bond pocket (D) and hydrophobic pocket (E) of IA into domain V-Vii of the catalytic site and domain V-Vii-specific residues of APN.

• Figure S13. 3D molecular binding pattern and 3D molecular interacting pattern of sildenafil into the catalytic site of PDE5.

• Figure S14. 3D molecular binding pattern and 3D molecular interacting pattern of bestatin

into the catalytic site of APN.

• Figure S15. The docking energy of vina analysis and 3D molecular binding pattern between ligand (DATS) and protein (PDE5 enzyme).

• Figure S16. The docking energy of vina analysis and 3D molecular binding pattern between ligand (DADS) and protein (PDE5 enzyme).

• Figure S17. The docking energy of vina analysis and 3D molecular binding pattern between ligand (KAE) and protein (PDE5 enzyme).

• Figure S18. The docking energy of vina analysis and 3D molecular binding pattern between ligand (QUE) and protein (PDE5 enzyme).

• Figure S19. The docking energy of vina analysis and 3D molecular binding pattern between ligand (MT) and protein (PDE5 enzyme).

• Figure S20. The docking energy of vina analysis and 3D molecular binding pattern between ligand (IA) and protein (PDE5 enzyme).

• Figure S21. The docking energy of vina analysis and 3D molecular binding pattern between ligand (DATS) and protein (APN enzyme).

• Figure S22. The docking energy of vina analysis and 3D molecular binding pattern between ligand (DADS) and protein (APN enzyme).

• Figure S23. The docking energy of vina analysis and 3D molecular binding pattern between ligand (KAE) and protein (APN enzyme).

• Figure S24. The docking energy of vina analysis and 3D molecular binding pattern between ligand (QUE) and protein (APN enzyme).

• Figure S25. The docking energy of vina analysis and 3D molecular binding pattern between ligand (MT) and protein (APN enzyme).

• Figure S26. The docking energy of vina analysis and 3D molecular binding pattern between ligand (IA) and protein (APN enzyme).

Figure S1

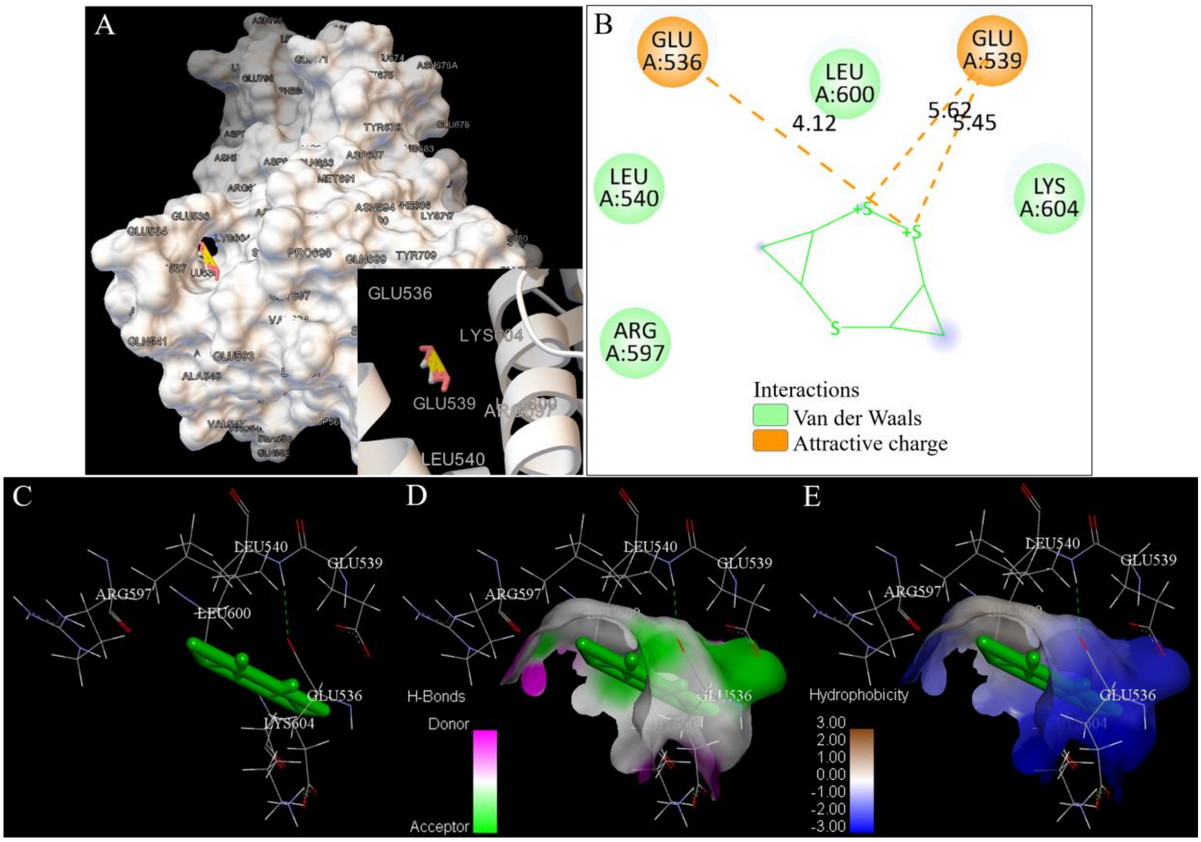
A
GLU536
LYS604
GLU539
ARG597
LEU540
B
GLU A:536
LEU A:600
GLU A:539
4.12
5.62
5.45
LEU A:540
LYS A:604
ARG A:597
Interactions
Van der Waals
Attractive charge
C
D
E
LEU540
GLU539
ARG597
LEU600
GLU536
LYS604
H-Bonds
Donor
Acceptor
Hydrophobicity
3.00
2.00
1.00
0.00
-1.00
-2.00
-3.00

Figure S2

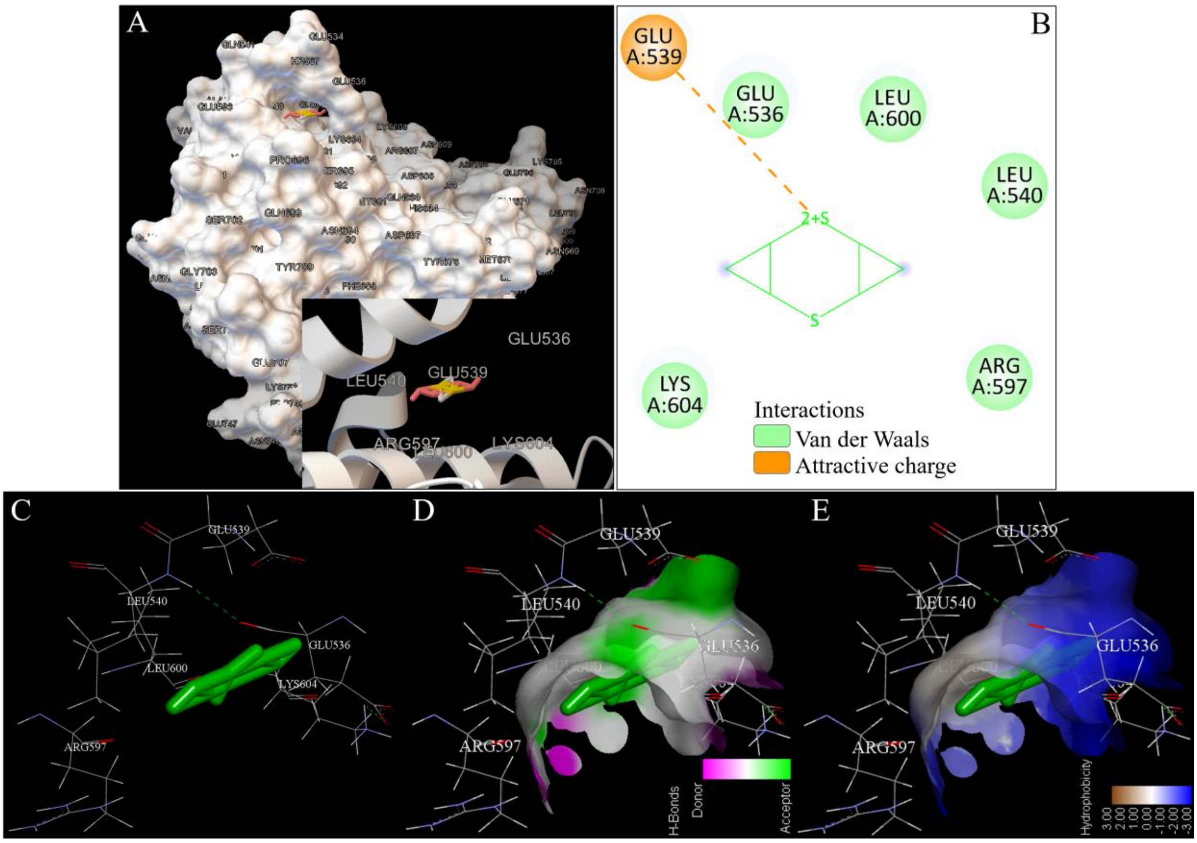
A
GLU536
LEU540
GLU539
ARG597
LYS604
B
GLU A:539
GLU A:536
LEU A:600
LEU A:540
2+S
S
LYS A:604
ARG A:597
Interactions
Van der Waals
Attractive charge
C
GLU539
LEU540
GLU536
LEU600
LYS604
ARG597
D
GLU539
LEU540
GLU536
ARG597
H-Bonds
Donor
Acceptor
E
GLU539
LEU540
GLU536
ARG597
Hydrophobicity
3.00
2.00
1.00
0.00
-1.00
-2.00
-3.00

Figure S3

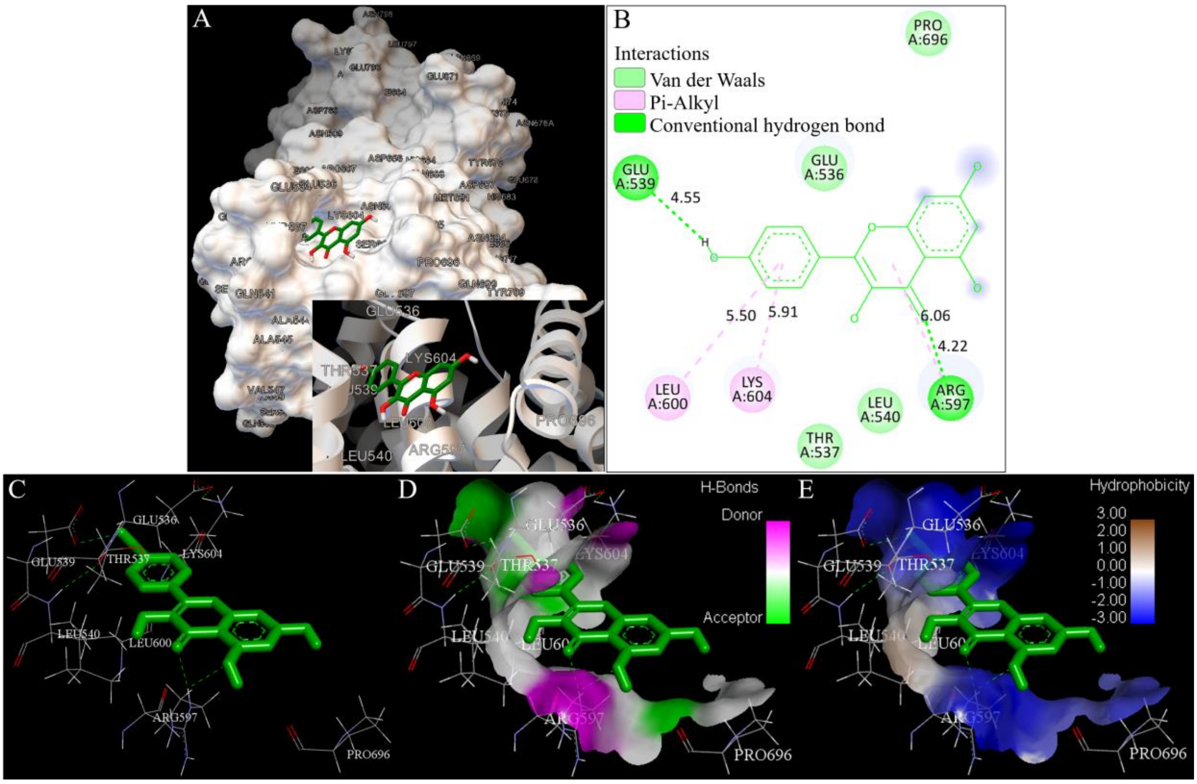

A
B
Interactions
Van der Waals
Pi-Alkyl
Conventional hydrogen bond
PRO A:696
GLU A:539
GLU A:536
4.55
5.50
5.91
6.06
4.22
LEU A:600
LYS A:604
LEU A:540
ARG A:597
THR A:537
C
D
E
H-Bonds
Donor
Acceptor
Hydrophobicity
3.00
2.00
1.00
0.00
-1.00
-2.00
-3.00
GLU536
GLU539
THR537
LYS604
LEU540
LEU600
ARG597
PRO696

Figure S4

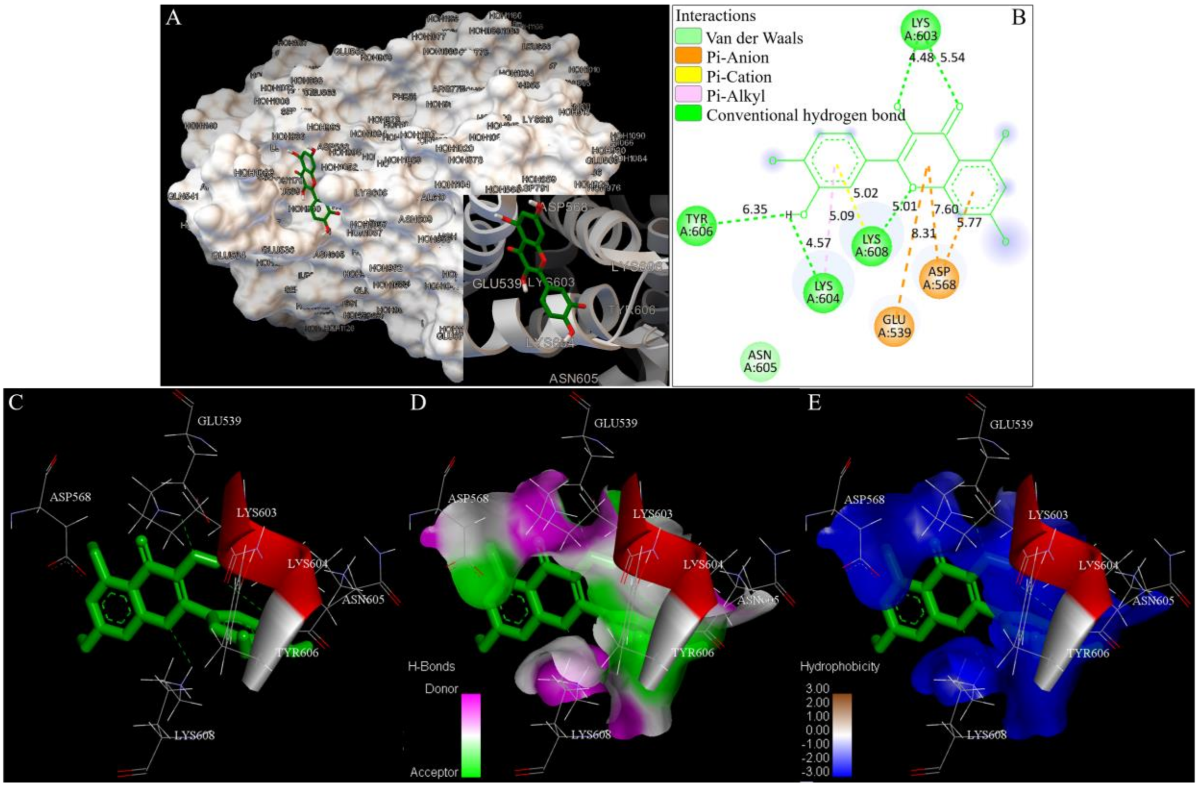

A
GLU539
LYS603
ASP568
TYR606
ASN605
B
Interactions
Van der Waals
Pi-Anion
Pi-Cation
Pi-Alkyl
Conventional hydrogen bond
LYS A:603
4.48
5.54
5.02
5.09
5.01
7.60
6.35
4.57
8.31
5.77
TYR A:606
LYS A:608
LYS A:604
ASP A:568
GLU A:539
ASN A:605
C
GLU539
ASP568
LYS603
LYS604
ASN605
TYR606
LYS608
D
GLU539
ASP568
LYS603
LYS604
ASN605
TYR606
LYS608
H-Bonds
Donor
Acceptor
E
GLU539
ASP568
LYS603
LYS604
ASN605
TYR606
LYS608
Hydrophobicity
3.00
2.00
1.00
0.00
-1.00
-2.00
-3.00

Figure S5

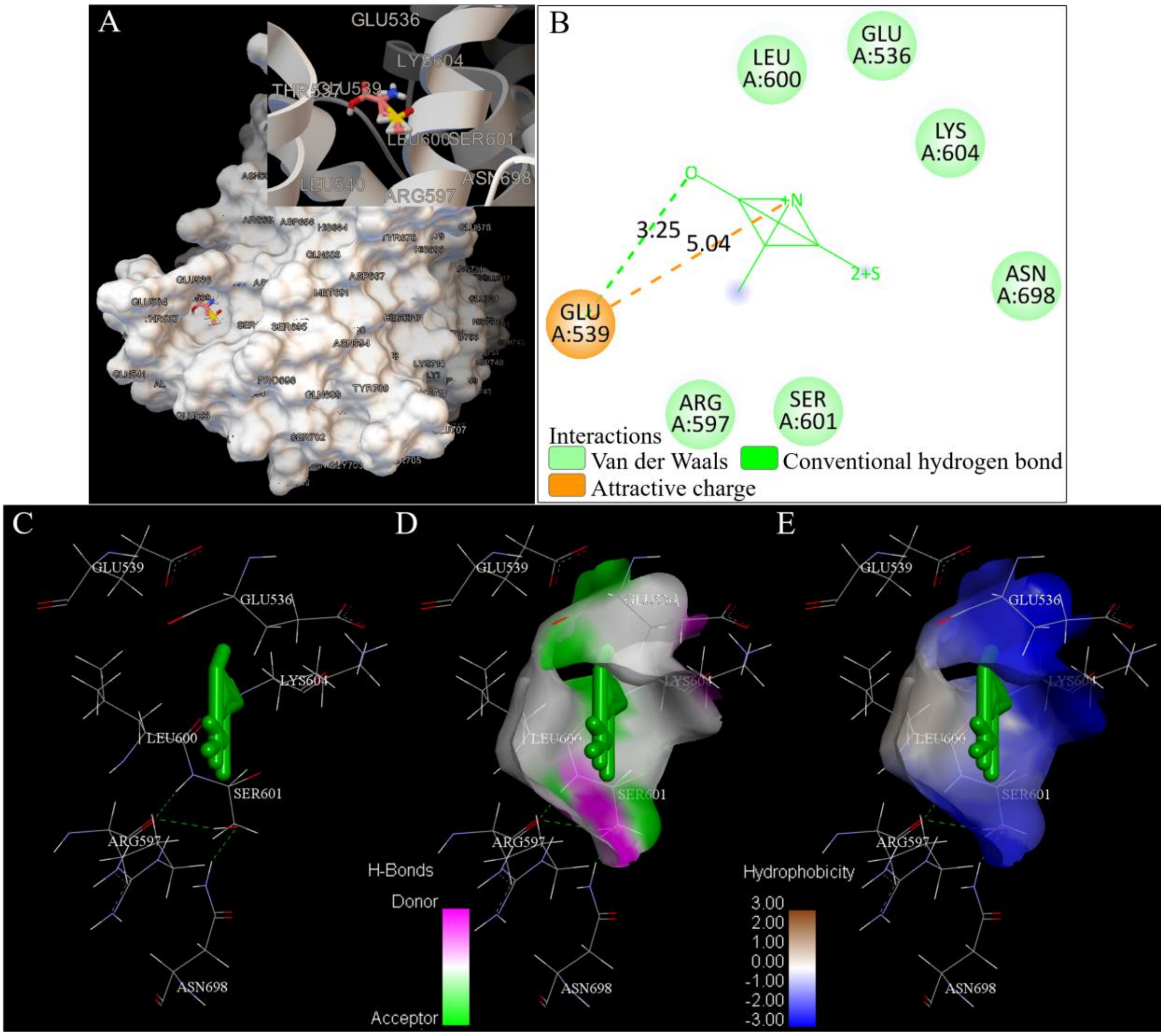
A
B
LEU
A:600
GLU
A:536
LYS
A:604
3.25
5.04
GLU
A:539
ASN
A:698
ARG
A:597
SER
A:601
Interactions
Van der Waals
Conventional hydrogen bond
Attractive charge
C
D
E
GLU539
GLU536
LYS604
LEU600
SER601
ARG597
ASN698
H-Bonds
Donor
Acceptor
Hydrophobicity
3.00
2.00
1.00
0.00
-1.00
-2.00
-3.00

Figure S6

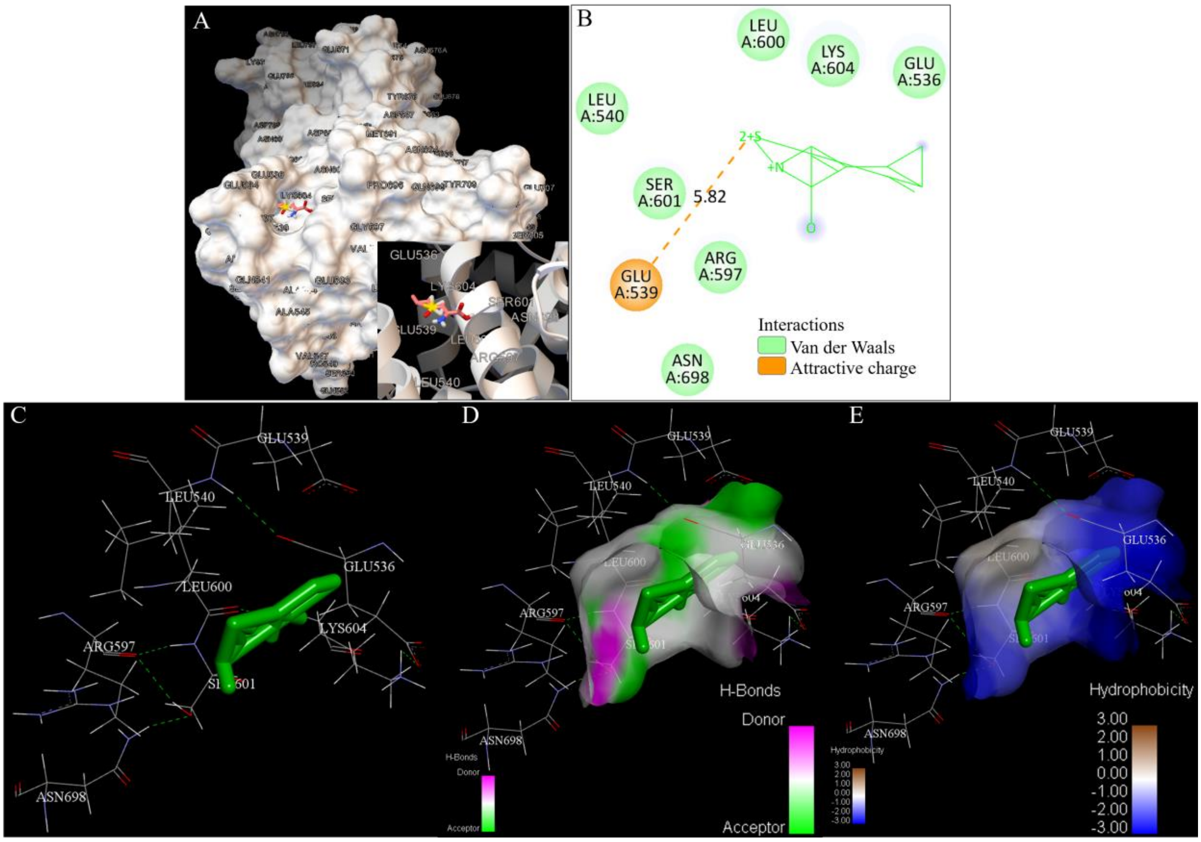

A
B
LEU A:600
LYS A:604
GLU A:536
LEU A:540
SER A:601
5.82
ARG A:597
GLU A:539
ASN A:698
Interactions
Van der Waals
Attractive charge
C
GLU539
LEU540
GLU536
LEU600
LYS604
ARG597
ASN698
D
H-Bonds
Donor
Acceptor
E
Hydrophobicity
3.00
2.00
1.00
0.00
-1.00
-2.00
-3.00

Figure S7

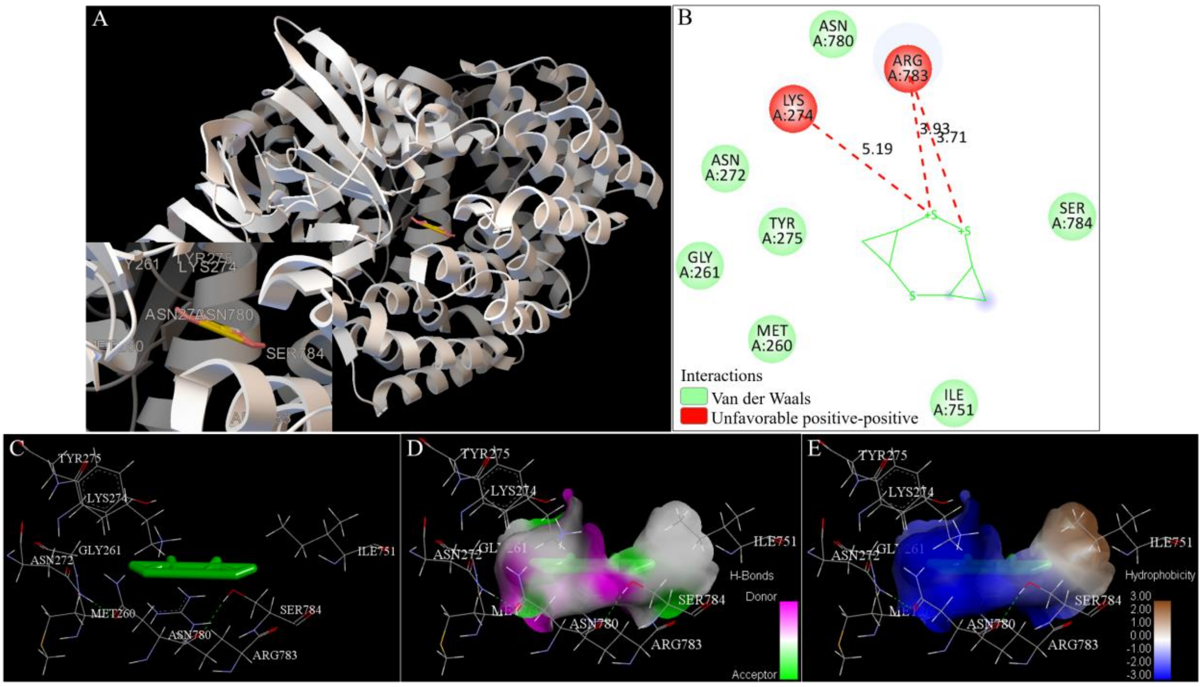
A
B
ASN
A:780
ARG
A:783
LYS
A:274
3.93
3.71
5.19
ASN
A:272
TYR
A:275
SER
A:784
GLY
A:261
MET
A:260
Interactions
Van der Waals
Unfavorable positive-positive
ILE
A:751
C
D
E
TYR275
LYS274
GLY261
ASN272
ILE751
MET260
SER784
ASN780
ARG783
H-Bonds
Donor
Acceptor
Hydrophobicity
3.00
2.00
1.00
0.00
-1.00
-2.00
-3.00

Figure S8

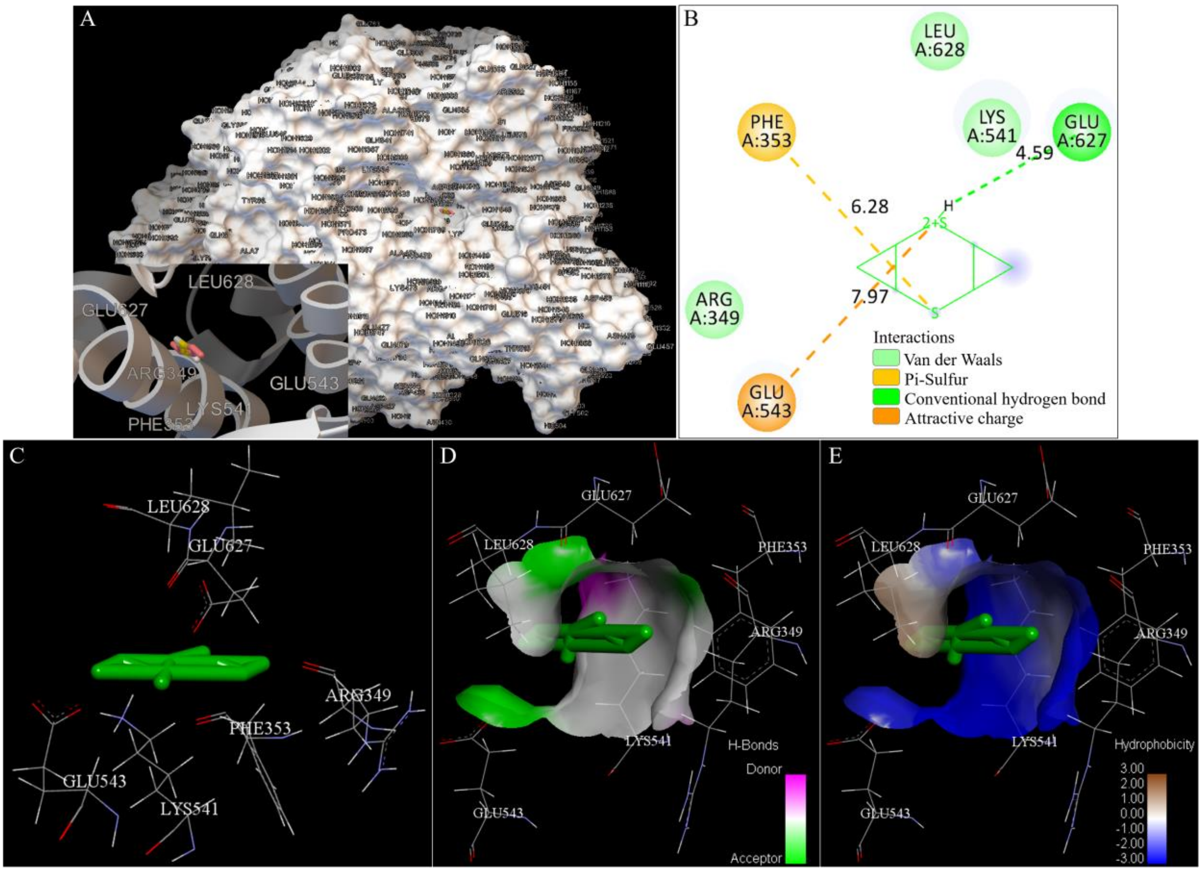
A
LEU628
GLU627
ARG349
GLU543
LYS541
PHE353
B
LEU A:628
PHE A:353
LYS A:541
GLU A:627
4.59
6.28
7.97
ARG A:349
GLU A:543
Interactions
Van der Waals
Pi-Sulfur
Conventional hydrogen bond
Attractive charge
C
LEU628
GLU627
ARG349
PHE353
GLU543
LYS541
D
GLU627
LEU628
PHE353
ARG349
LYS541
H-Bonds
Donor
Acceptor
GLU543
E
GLU627
LEU628
PHE353
ARG349
LYS541
Hydrophobicity
3.00
2.00
1.00
0.00
-1.00
-2.00
-3.00
GLU543

Figure S9

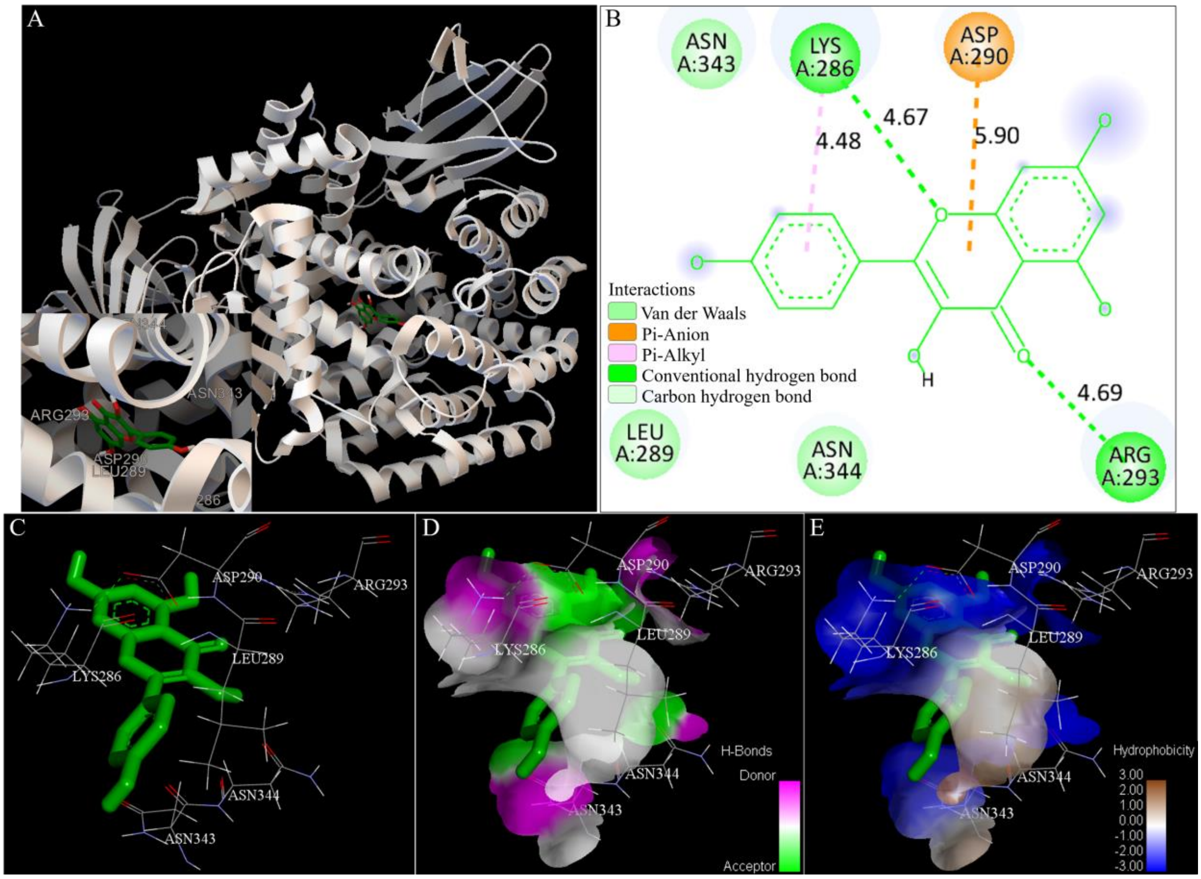
A
ARG293
ASN343
ASP290
LEU289
B
ASN A:343
LYS A:286
ASP A:290
4.67
4.48
5.90
4.69
H
Interactions
Van der Waals
Pi-Anion
Pi-Alkyl
Conventional hydrogen bond
Carbon hydrogen bond
LEU A:289
ASN A:344
ARG A:293
C
ASP290
ARG293
LEU289
LYS286
ASN344
ASN343
D
ASP290
ARG293
LEU289
LYS286
H-Bonds
Donor
ASN344
ASN343
Acceptor
E
ASP290
ARG293
LEU289
LYS286
Hydrophobicity
ASN344
ASN343
3.00
2.00
1.00
0.00
-1.00
-2.00
-3.00

Figure S10

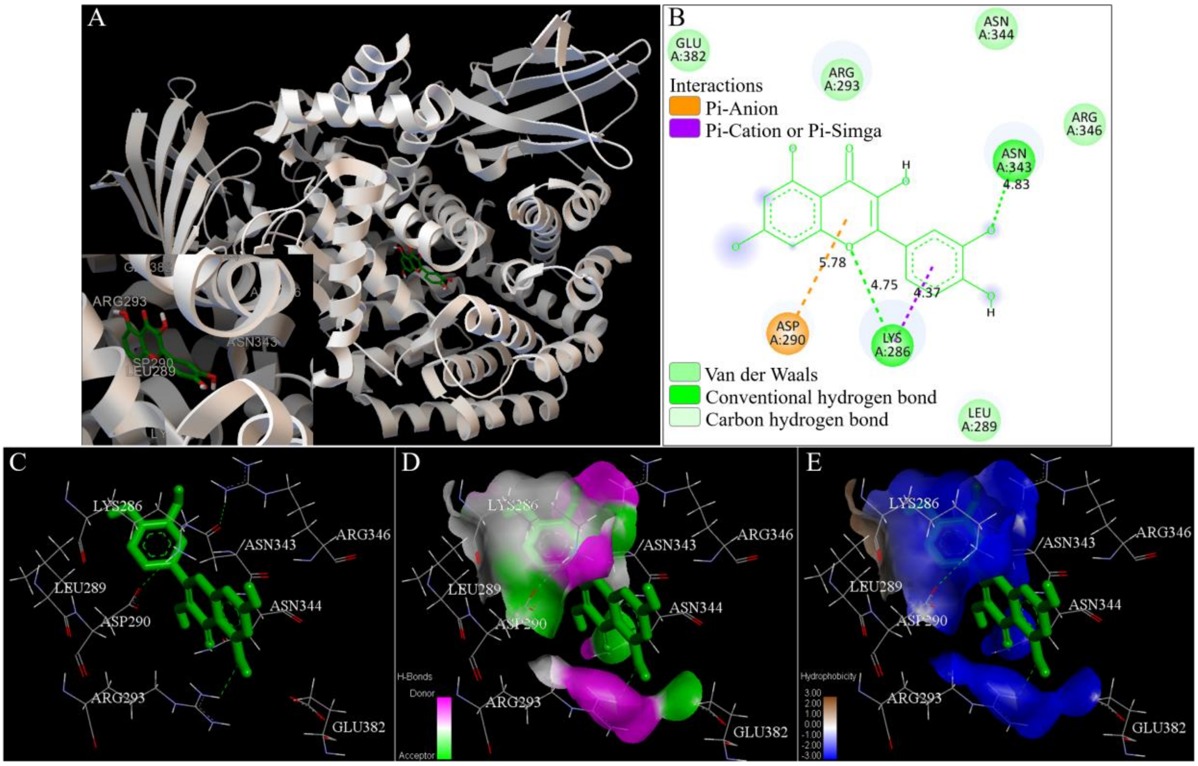
A
ARG293
ASN343
ASP290
LEU289
B
GLU A:382
ARG A:293
ASN A:344
ARG A:346
ASN A:343
Interactions
Pi-Anion
Pi-Cation or Pi-Simga
4.83
5.78
4.75
4.37
ASP A:290
LYS A:286
Van der Waals
Conventional hydrogen bond
Carbon hydrogen bond
LEU A:289
C
LYS286
ASN343
ARG346
LEU289
ASP290
ASN344
ARG293
GLU382
D
H-Bonds
Donor
Acceptor
E
Hydrophobicity
3.00
2.00
1.00
0.00
-1.00
-2.00
-3.00

Figure S11

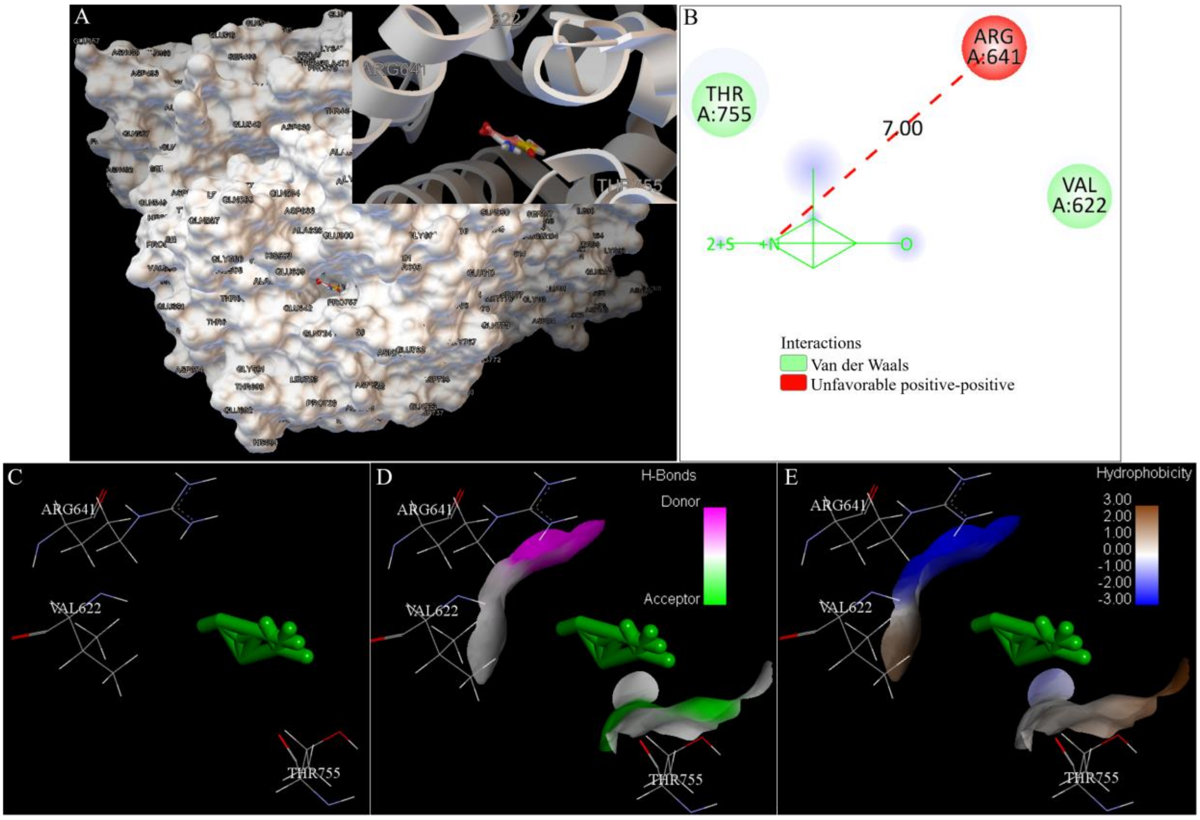

A
B
ARG
A:641
THR
A:755
7.00
VAL
A:622
Interactions
Van der Waals
Unfavorable positive-positive
C
ARG641
VAL622
THR755
D
H-Bonds
Donor
Acceptor
ARG641
VAL622
THR755
E
Hydrophobicity
3.00
2.00
1.00
0.00
-1.00
-2.00
-3.00
ARG641
VAL622
THR755

Figure S12

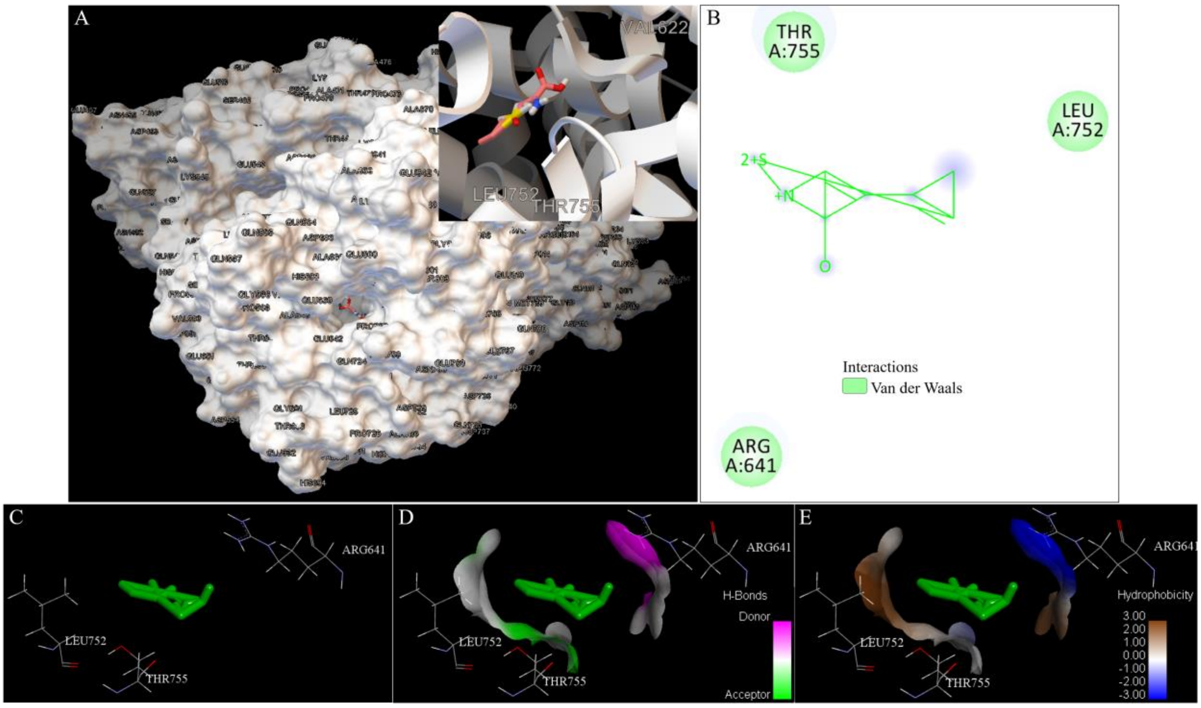

A
VAL622
LEU752
THR755
B
THR A:755
LEU A:752
ARG A:641
Interactions
Van der Waals
C
ARG641
LEU752
THR755
D
ARG641
H-Bonds
Donor
Acceptor
LEU752
THR755
E
ARG641
Hydrophobicity
3.00
2.00
1.00
0.00
-1.00
-2.00
-3.00
LEU752
THR755

Figure S13

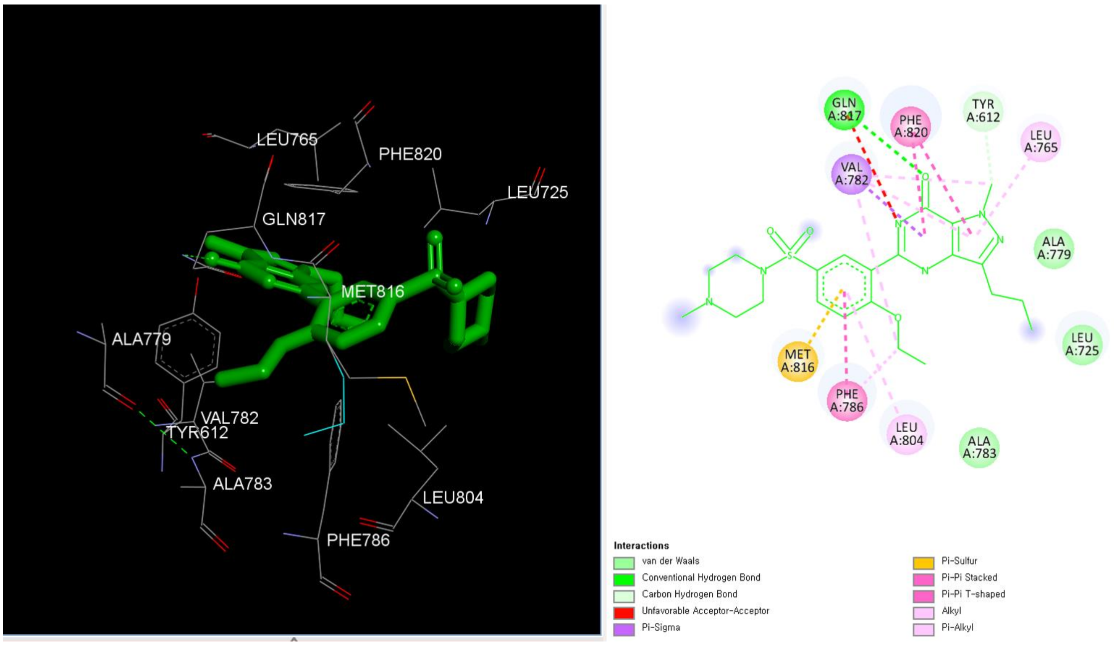
LEU765
PHE820
LEU725
GLN817
MET816
ALA779
VAL782
TYR612
ALA783
LEU804
PHE786
GLN A:817
PHE A:820
TYR A:612
LEU A:765
VAL A:782
ALA A:779
LEU A:725
MET A:816
PHE A:786
LEU A:804
ALA A:783
Interactions
van der Waals
Conventional Hydrogen Bond
Carbon Hydrogen Bond
Unfavorable Acceptor-Acceptor
Pi-Sigma
Pi-Sulfur
Pi-Pi Stacked
Pi-Pi T-shaped
Alkyl
Pi-Alkyl

Figure S14

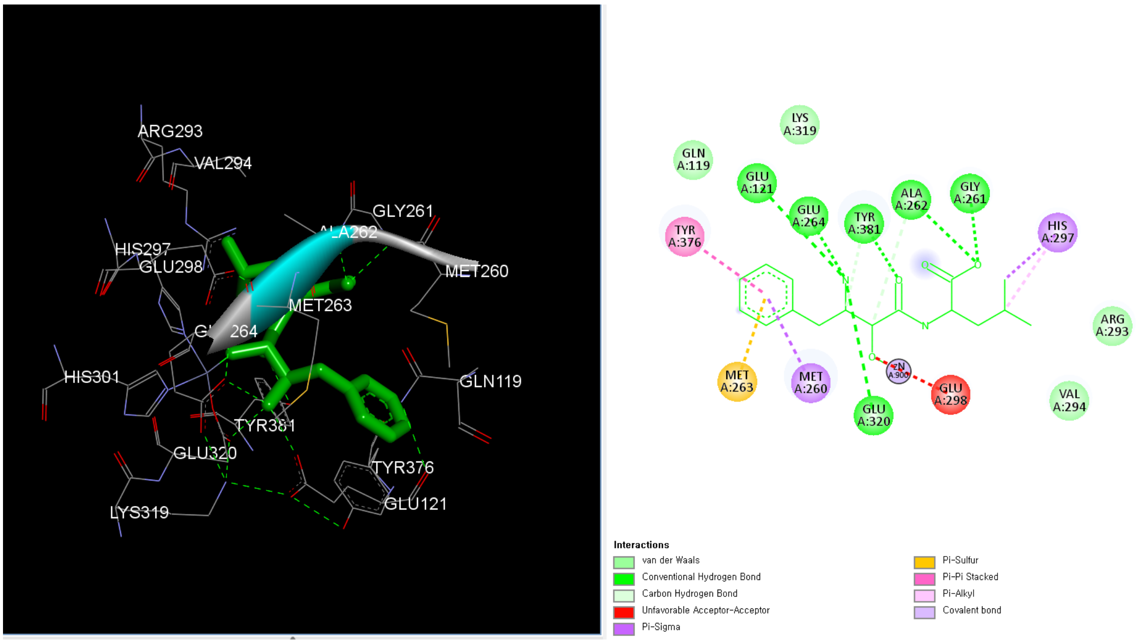
ARG293
VAL294
GLY261
HIS297
GLU298
MET260
MET263
HIS301
GLN119
TYR381
GLU320
TYR376
LYS319
GLU121
LYS A:319
GLN A:119
GLU A:121
GLU A:264
TYR A:381
ALA A:262
GLY A:261
TYR A:376
HIS A:297
ARG A:293
MET A:263
MET A:260
GLU A:320
GLU A:298
VAL A:294
Interactions
van der Waals
Conventional Hydrogen Bond
Carbon Hydrogen Bond
Unfavorable Acceptor-Acceptor
Pi-Sigma
Pi-Sulfur
Pi-Pi Stacked
Pi-Alkyl
Covalent bond

Figure S15

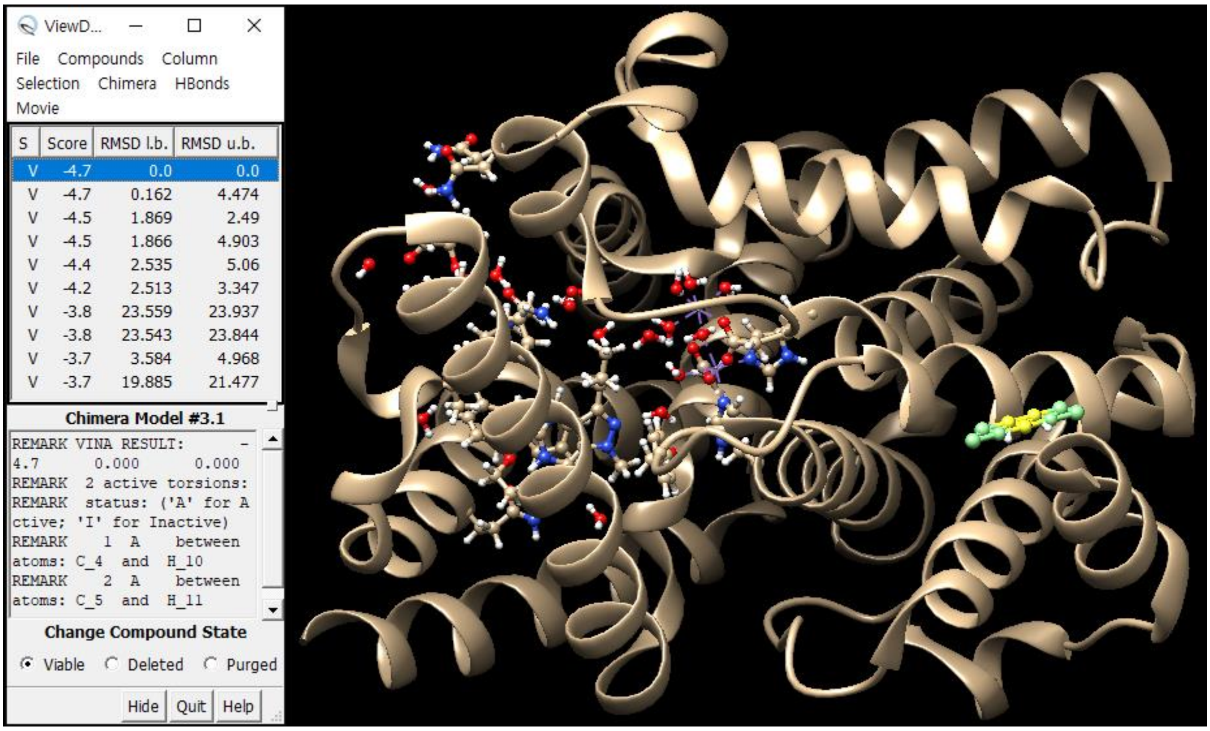
ViewD...
File Compounds Column
Selection Chimera HBonds
Movie
S Score RMSD l.b. RMSD u.b.
V -4.7 0.0 0.0
V -4.7 0.162 4.474
V -4.5 1.869 2.49
V -4.5 1.866 4.903
V -4.4 2.535 5.06
V -4.2 2.513 3.347
V -3.8 23.559 23.937
V -3.8 23.543 23.844
V -3.7 3.584 4.968
V -3.7 19.885 21.477
Chimera Model #3.1
REMARK VINA RESULT: -4.7 0.000 0.000
REMARK 2 active torsions:
REMARK status: ('A' for Active; 'I' for Inactive)
REMARK 1 A between atoms: C_4 and H_10
REMARK 2 A between atoms: C_5 and H_11
Change Compound State
Viable Deleted Purged
Hide Quit Help

Figure S16

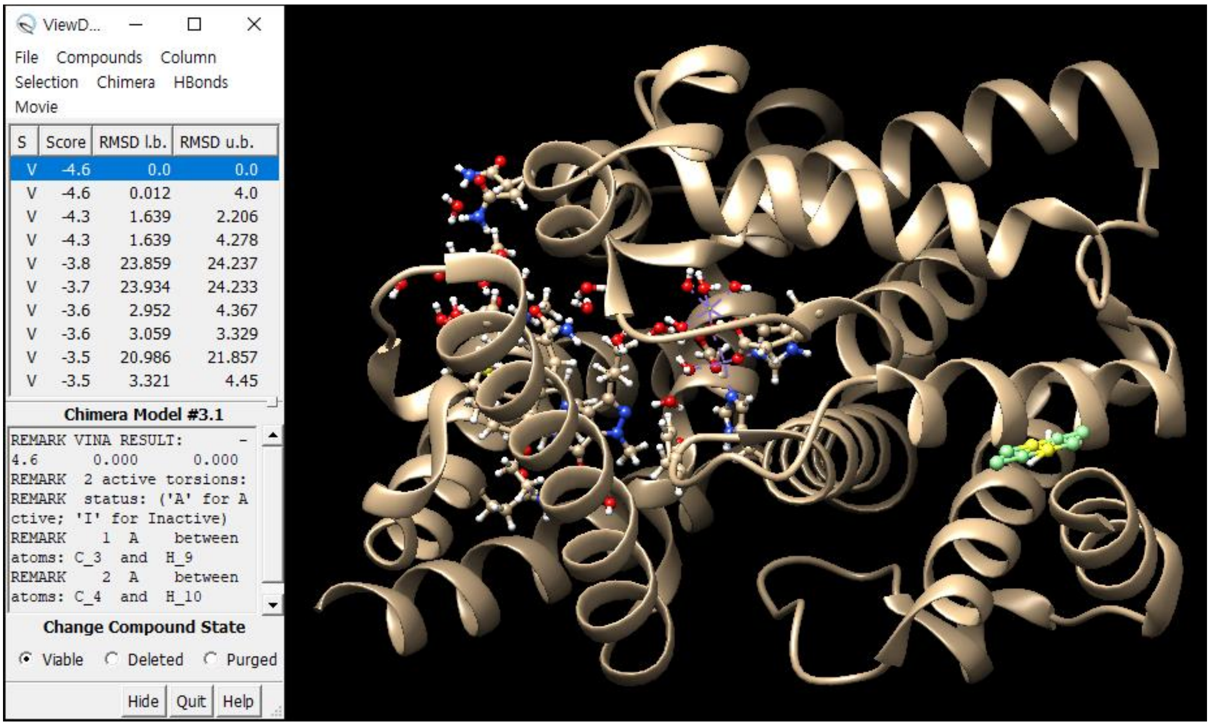
ViewD...
File Compounds Column
Selection Chimera HBonds
Movie
S Score RMSD l.b. RMSD u.b.
V -4.6 0.0 0.0
V -4.6 0.012 4.0
V -4.3 1.639 2.206
V -4.3 1.639 4.278
V -3.8 23.859 24.237
V -3.7 23.934 24.233
V -3.6 2.952 4.367
V -3.6 3.059 3.329
V -3.5 20.986 21.857
V -3.5 3.321 4.45
Chimera Model #3.1
REMARK VINA RESULT: -4.6 0.000 0.000
REMARK 2 active torsions:
REMARK status: ('A' for Active; 'I' for Inactive)
REMARK 1 A between atoms: C_3 and H_9
REMARK 2 A between atoms: C_4 and H_10
Change Compound State
Viable Deleted Purged
Hide Quit Help

Figure S17

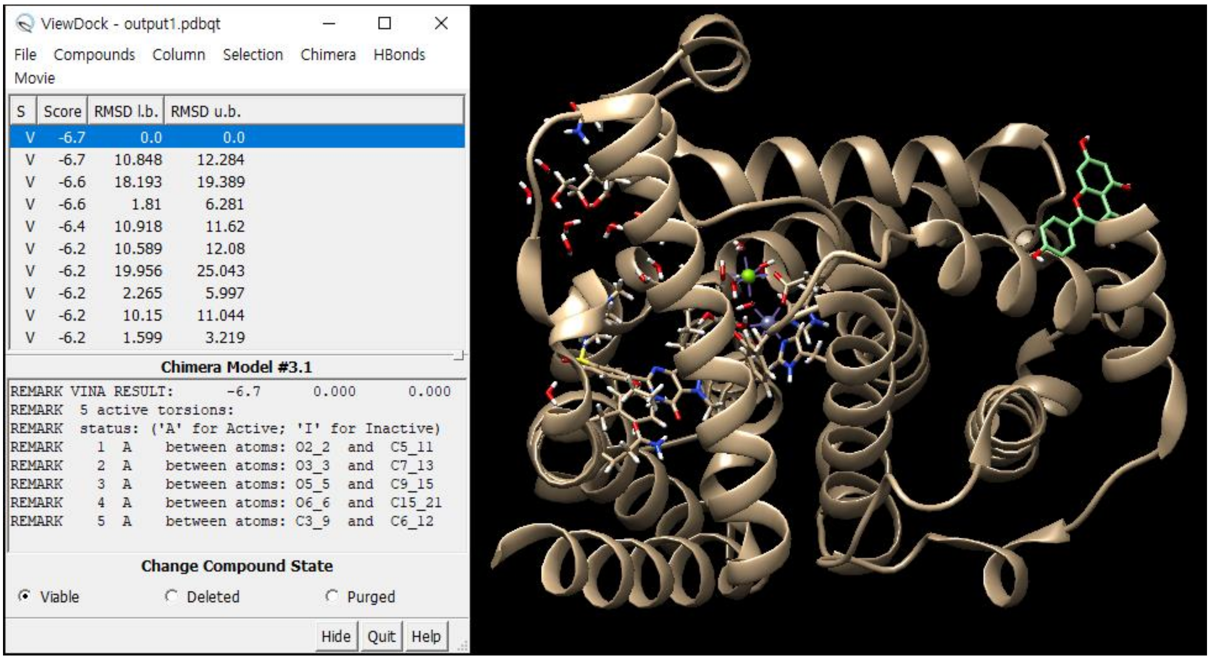
ViewDock - output1.pdbqt
File Compounds Column Selection Chimera HBonds
Movie
S Score RMSD l.b. RMSD u.b.
V -6.7 0.0 0.0
V -6.7 10.848 12.284
V -6.6 18.193 19.389
V -6.6 1.81 6.281
V -6.4 10.918 11.62
V -6.2 10.589 12.08
V -6.2 19.956 25.043
V -6.2 2.265 5.997
V -6.2 10.15 11.044
V -6.2 1.599 3.219
Chimera Model #3.1
REMARK VINA RESULT: -6.7 0.000 0.000
REMARK 5 active torsions:
REMARK status: ('A' for Active; 'I' for Inactive)
REMARK 1 A between atoms: O2_2 and C5_11
REMARK 2 A between atoms: O3_3 and C7_13
REMARK 3 A between atoms: O5_5 and C9_15
REMARK 4 A between atoms: O6_6 and C15_21
REMARK 5 A between atoms: C3_9 and C6_12
Change Compound State
Viable
Deleted
Purged
Hide
Quit
Help

Figure S18

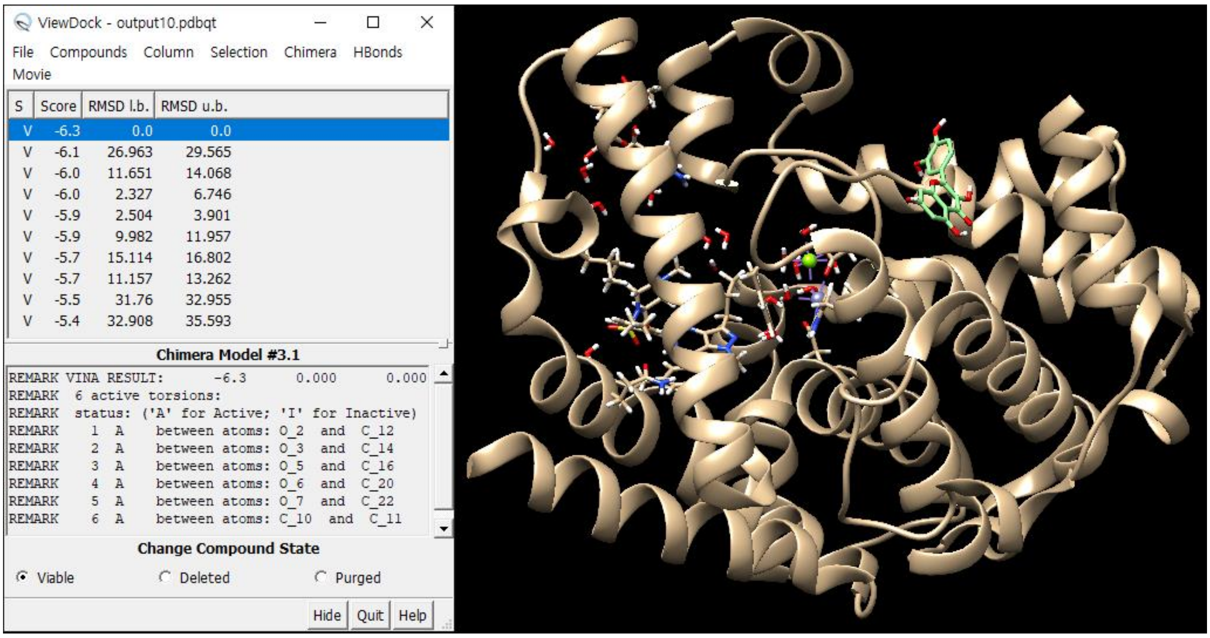

ViewDock - output10.pdbqt
File Compounds Column Selection Chimera HBonds
Movie
S Score RMSD l.b. RMSD u.b.
V -6.3 0.0 0.0
V -6.1 26.963 29.565
V -6.0 11.651 14.068
V -6.0 2.327 6.746
V -5.9 2.504 3.901
V -5.9 9.982 11.957
V -5.7 15.114 16.802
V -5.7 11.157 13.262
V -5.5 31.76 32.955
V -5.4 32.908 35.593
Chimera Model #3.1
REMARK VINA RESULT: -6.3 0.000 0.000
REMARK 6 active torsions:
REMARK status: ('A' for Active; 'I' for Inactive)
REMARK 1 A between atoms: O_2 and C_12
REMARK 2 A between atoms: O_3 and C_14
REMARK 3 A between atoms: O_5 and C_16
REMARK 4 A between atoms: O_6 and C_20
REMARK 5 A between atoms: O_7 and C_22
REMARK 6 A between atoms: C_10 and C_11
Change Compound State
Viable
Deleted
Purged
Hide
Quit
Help

Figure S19

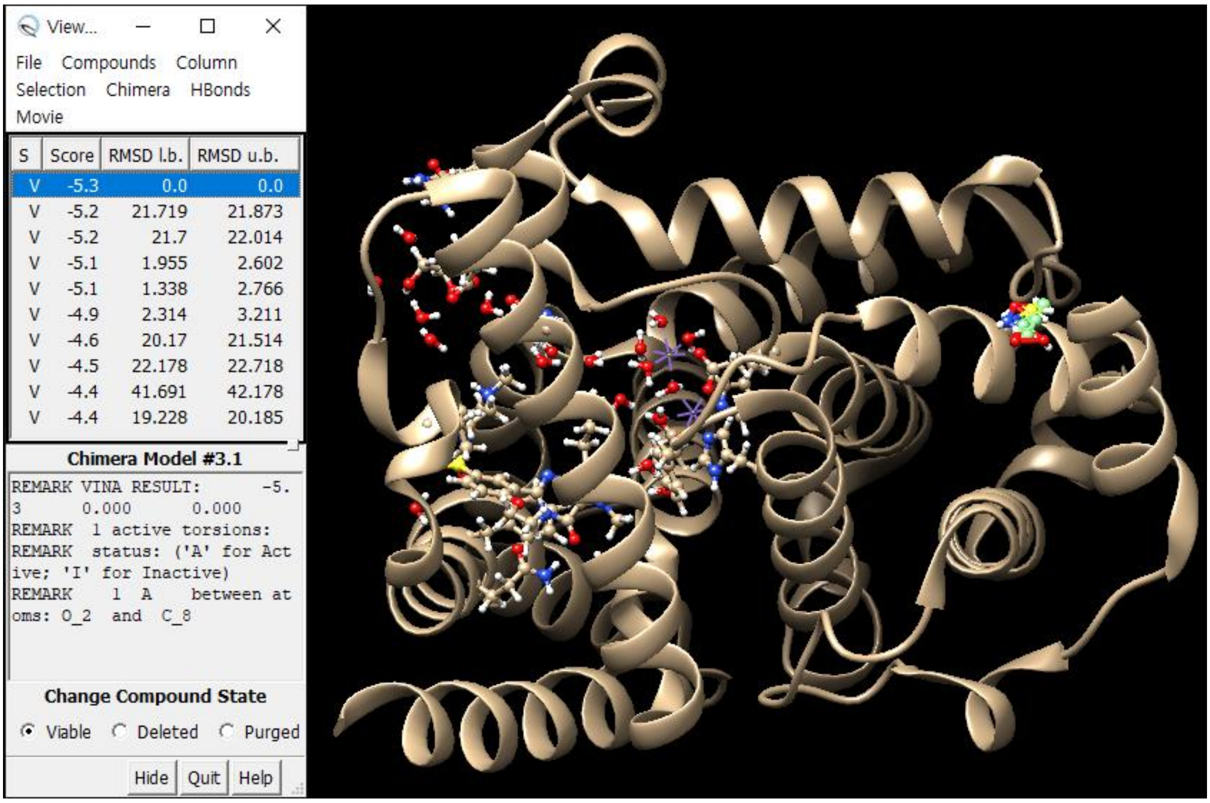
View...
File Compounds Column
Selection Chimera HBonds
Movie
S Score RMSD l.b. RMSD u.b.
V -5.3 0.0 0.0
V -5.2 21.719 21.873
V -5.2 21.7 22.014
V -5.1 1.955 2.602
V -5.1 1.338 2.766
V -4.9 2.314 3.211
V -4.6 20.17 21.514
V -4.5 22.178 22.718
V -4.4 41.691 42.178
V -4.4 19.228 20.185
Chimera Model #3.1
REMARK VINA RESULT: -5.3 0.000 0.000
REMARK 1 active torsions:
REMARK status: ('A' for Active; 'I' for Inactive)
REMARK 1 A between atoms: O_2 and C_8
Change Compound State
Viable Deleted Purged
Hide Quit Help

Figure S20

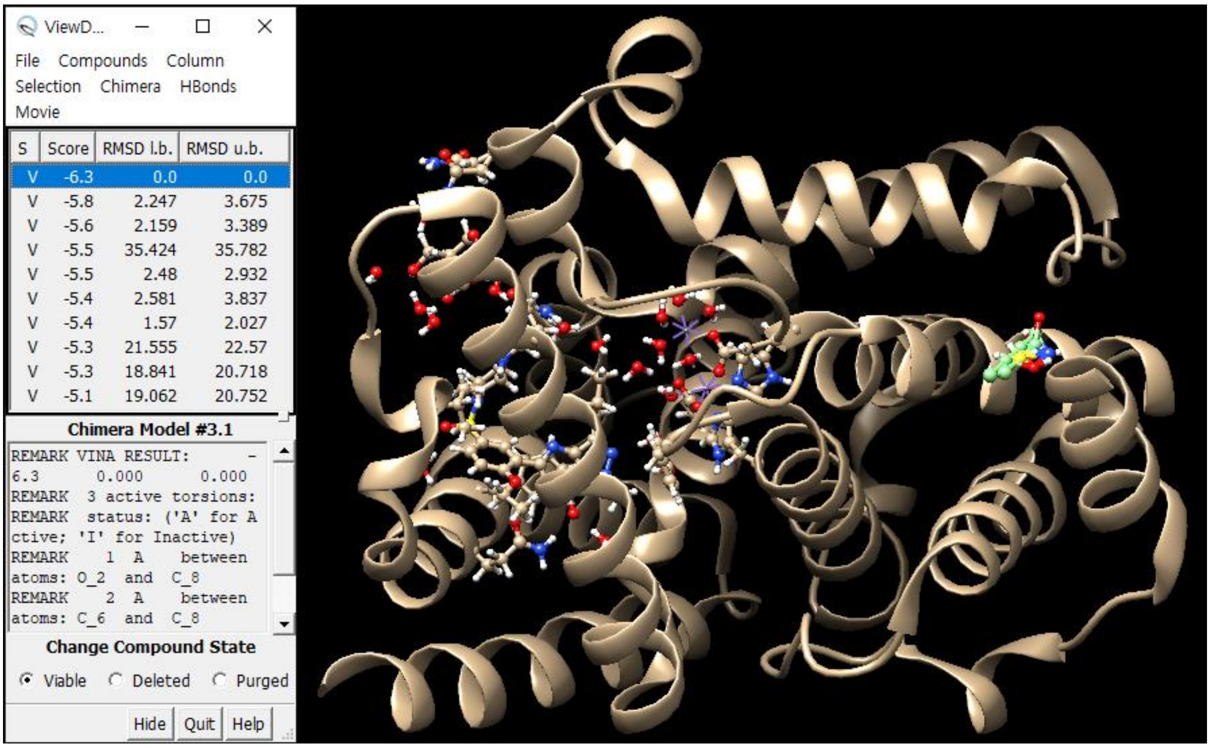
ViewD...
File Compounds Column
Selection Chimera HBonds
Movie
S | Score | RMSD l.b. | RMSD u.b.
V -6.3 0.0 0.0
V -5.8 2.247 3.675
V -5.6 2.159 3.389
V -5.5 35.424 35.782
V -5.5 2.48 2.932
V -5.4 2.581 3.837
V -5.4 1.57 2.027
V -5.3 21.555 22.57
V -5.3 18.841 20.718
V -5.1 19.062 20.752
Chimera Model #3.1
REMARK VINA RESULT: -
6.3 0.000 0.000
REMARK 3 active torsions:
REMARK status: ('A' for A
ctive; 'I' for Inactive)
REMARK 1 A between
atoms: O_2 and C_8
REMARK 2 A between
atoms: C_6 and C_8
Change Compound State
Viable Deleted Purged
Hide Quit Help

Figure S21

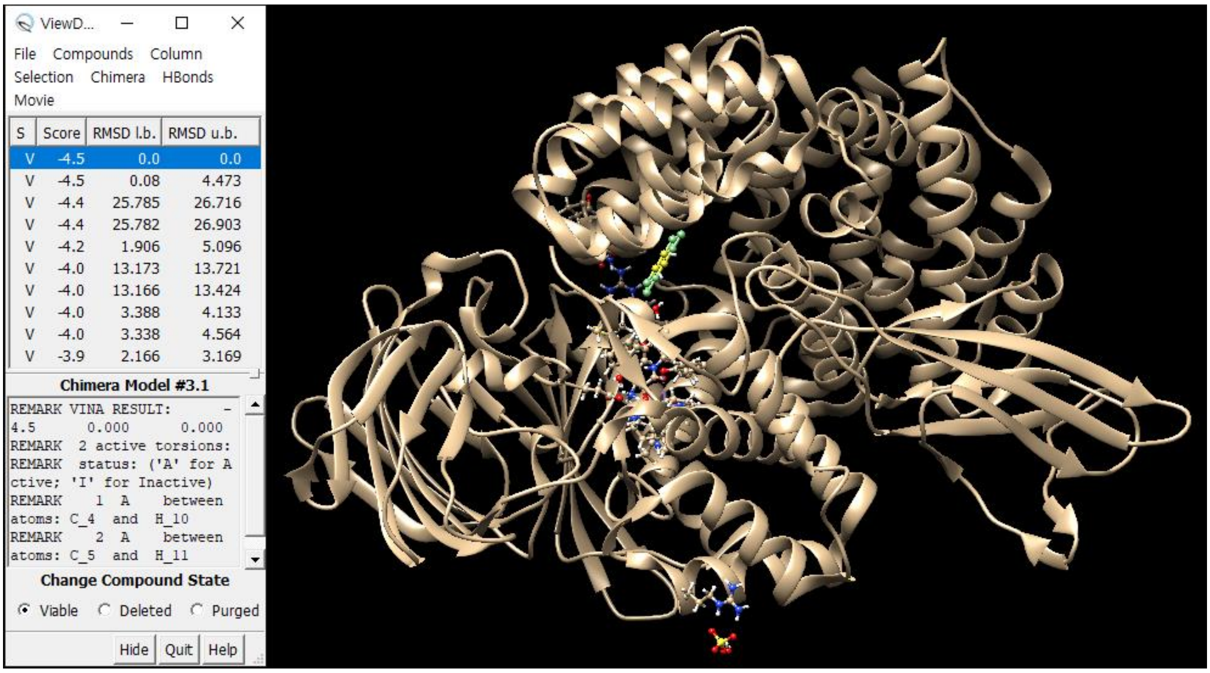
ViewD...
File Compounds Column
Selection Chimera HBonds
Movie
S Score RMSD l.b. RMSD u.b.
V -4.5 0.0 0.0
V -4.5 0.08 4.473
V -4.4 25.785 26.716
V -4.4 25.782 26.903
V -4.2 1.906 5.096
V -4.0 13.173 13.721
V -4.0 13.166 13.424
V -4.0 3.388 4.133
V -4.0 3.338 4.564
V -3.9 2.166 3.169
Chimera Model #3.1
REMARK VINA RESULT: -4.5 0.000 0.000
REMARK 2 active torsions:
REMARK status: ('A' for Active; 'I' for Inactive)
REMARK 1 A between atoms: C_4 and H_10
REMARK 2 A between atoms: C_5 and H_11
Change Compound State
Viable Deleted Purged
Hide Quit Help

Figure S22

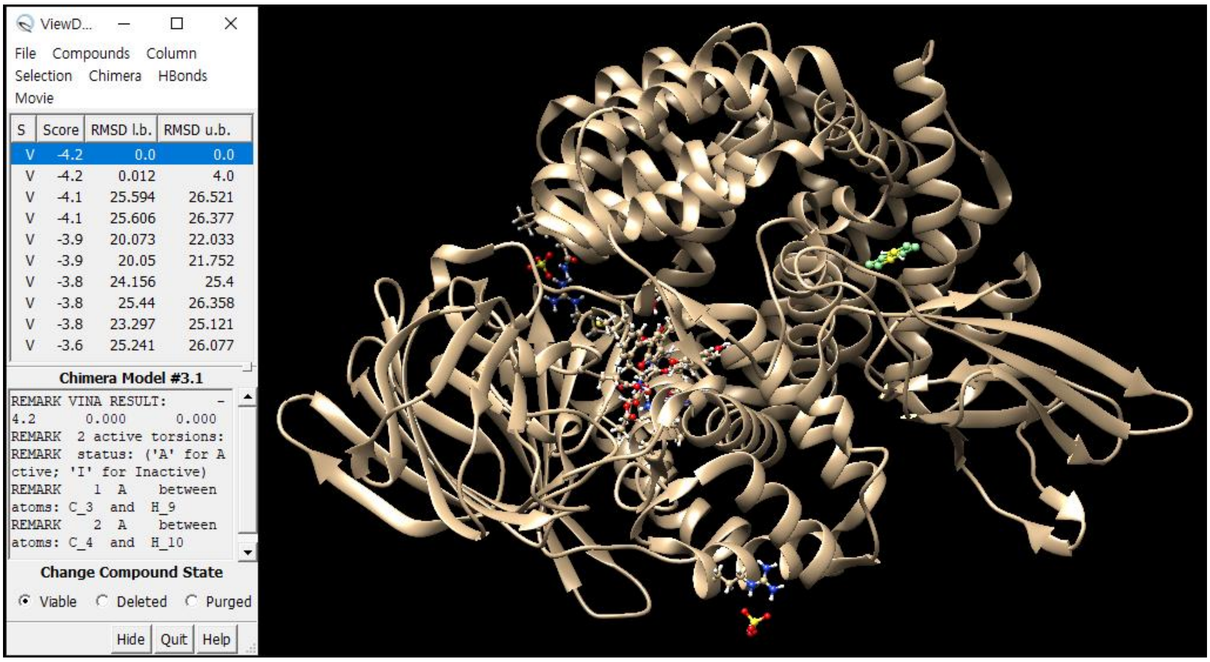
ViewD...
File Compounds Column
Selection Chimera HBonds
Movie
S Score RMSD l.b. RMSD u.b.
V -4.2 0.0 0.0
V -4.2 0.012 4.0
V -4.1 25.594 26.521
V -4.1 25.606 26.377
V -3.9 20.073 22.033
V -3.9 20.05 21.752
V -3.8 24.156 25.4
V -3.8 25.44 26.358
V -3.8 23.297 25.121
V -3.6 25.241 26.077
Chimera Model #3.1
REMARK VINA RESULT: -4.2 0.000 0.000
REMARK 2 active torsions:
REMARK status: ('A' for Active; 'I' for Inactive)
REMARK 1 A between atoms: C_3 and H_9
REMARK 2 A between atoms: C_4 and H_10
Change Compound State
Viable Deleted Purged
Hide Quit Help

Figure S23

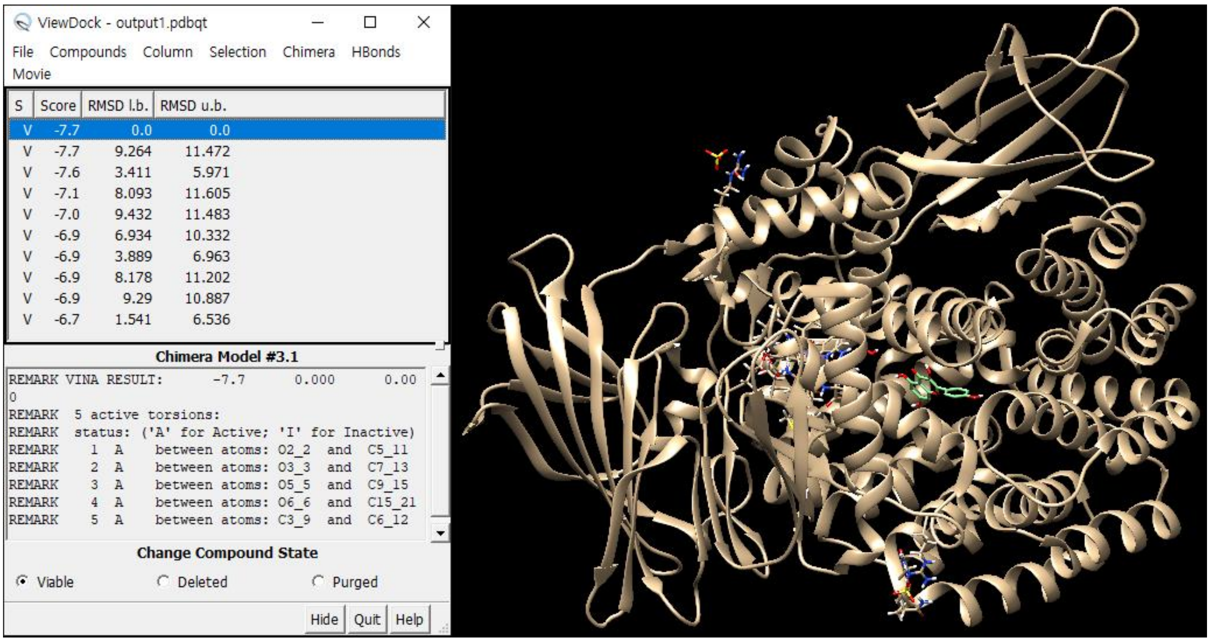
ViewDock - output1.pdbqt
File Compounds Column Selection Chimera HBonds
Movie
S Score RMSD l.b. RMSD u.b.
V -7.7 0.0 0.0
V -7.7 9.264 11.472
V -7.6 3.411 5.971
V -7.1 8.093 11.605
V -7.0 9.432 11.483
V -6.9 6.934 10.332
V -6.9 3.889 6.963
V -6.9 8.178 11.202
V -6.9 9.29 10.887
V -6.7 1.541 6.536
Chimera Model #3.1
REMARK VINA RESULT: -7.7 0.000 0.00
0
REMARK 5 active torsions:
REMARK status: ('A' for Active; 'I' for Inactive)
REMARK 1 A between atoms: O2_2 and C5_11
REMARK 2 A between atoms: O3_3 and C7_13
REMARK 3 A between atoms: O5_5 and C9_15
REMARK 4 A between atoms: O6_6 and C15_21
REMARK 5 A between atoms: C3_9 and C6_12
Change Compound State
Viable
Deleted
Purged
Hide
Quit
Help

Figure S24

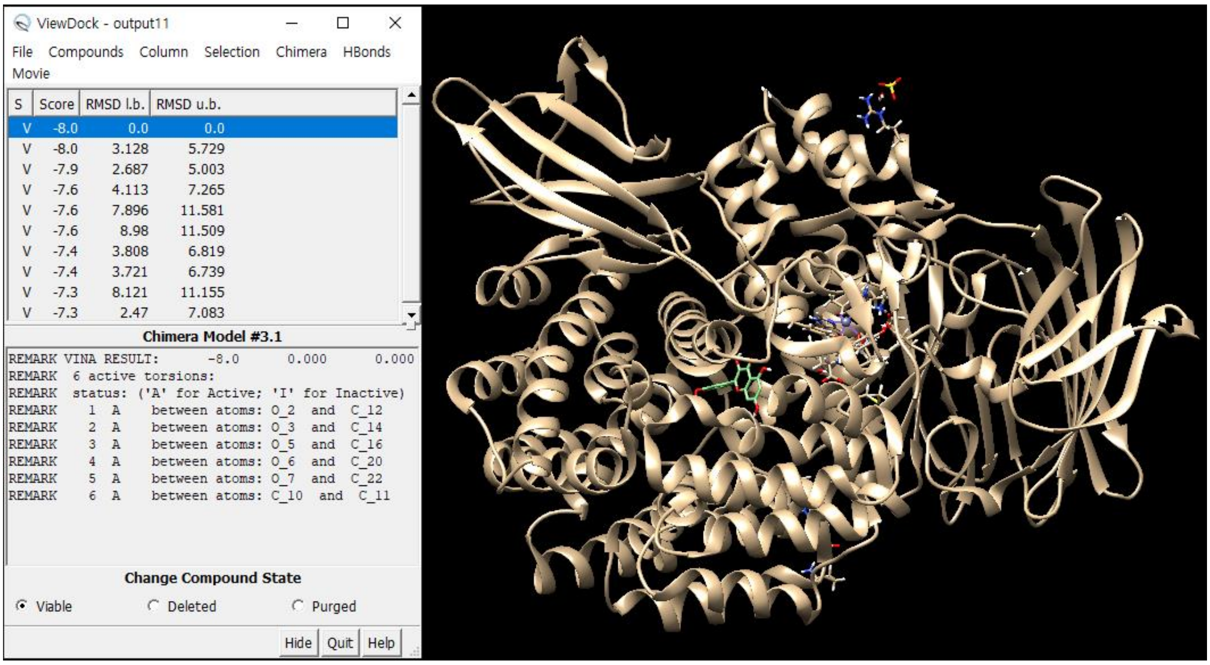
ViewDock - output11
File Compounds Column Selection Chimera HBonds
Movie
S Score RMSD l.b. RMSD u.b.
V -8.0 0.0 0.0
V -8.0 3.128 5.729
V -7.9 2.687 5.003
V -7.6 4.113 7.265
V -7.6 7.896 11.581
V -7.6 8.98 11.509
V -7.4 3.808 6.819
V -7.4 3.721 6.739
V -7.3 8.121 11.155
V -7.3 2.47 7.083
Chimera Model #3.1
REMARK VINA RESULT: -8.0 0.000 0.000
REMARK 6 active torsions:
REMARK status: ('A' for Active; 'I' for Inactive)
REMARK 1 A between atoms: O_2 and C_12
REMARK 2 A between atoms: O_3 and C_14
REMARK 3 A between atoms: O_5 and C_16
REMARK 4 A between atoms: O_6 and C_20
REMARK 5 A between atoms: O_7 and C_22
REMARK 6 A between atoms: C_10 and C_11
Change Compound State
Viable
Deleted
Purged
Hide
Quit
Help

Figure S25

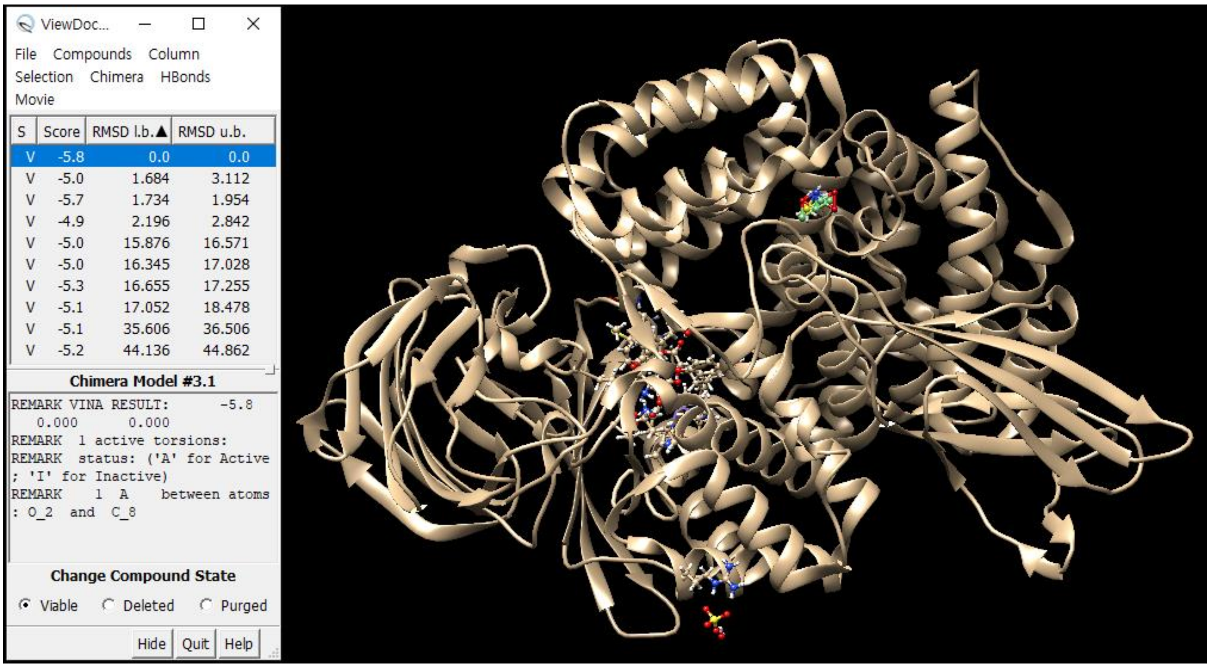
ViewDoc...
File Compounds Column
Selection Chimera HBonds
Movie
S Score RMSD l.b. RMSD u.b.
V -5.8 0.0 0.0
V -5.0 1.684 3.112
V -5.7 1.734 1.954
V -4.9 2.196 2.842
V -5.0 15.876 16.571
V -5.0 16.345 17.028
V -5.3 16.655 17.255
V -5.1 17.052 18.478
V -5.1 35.606 36.506
V -5.2 44.136 44.862
Chimera Model #3.1
REMARK VINA RESULT: -5.8 0.000 0.000
REMARK 1 active torsions:
REMARK status: ('A' for Active ; 'I' for Inactive)
REMARK 1 A between atoms : O_2 and C_8
Change Compound State
Viable Deleted Purged
Hide Quit Help

Figure S26

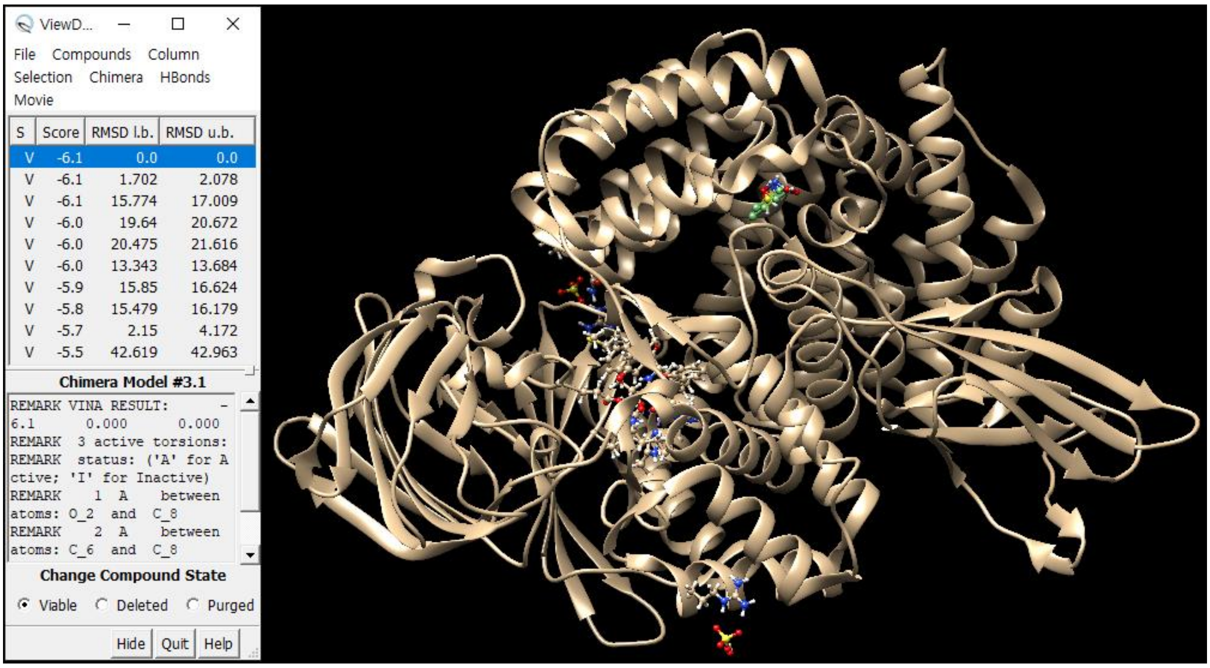
ViewD...
File Compounds Column
Selection Chimera HBonds
Movie
S Score RMSD l.b. RMSD u.b.
V -6.1 0.0 0.0
V -6.1 1.702 2.078
V -6.1 15.774 17.009
V -6.0 19.64 20.672
V -6.0 20.475 21.616
V -6.0 13.343 13.684
V -5.9 15.85 16.624
V -5.8 15.479 16.179
V -5.7 2.15 4.172
V -5.5 42.619 42.963
Chimera Model #3.1
REMARK VINA RESULT: -
6.1 0.000 0.000
REMARK 3 active torsions:
REMARK status: ('A' for A
ctive; 'I' for Inactive)
REMARK 1 A between
atoms: O_2 and C_8
REMARK 2 A between
atoms: C_6 and C_8
Change Compound State
Viable Deleted Purged
Hide Quit Help
